# Speeding Up Social Waves. Propagation Mechanisms of Shimmering in Giant Honeybees

**DOI:** 10.1371/journal.pone.0086315

**Published:** 2014-01-27

**Authors:** Gerald Kastberger, Thomas Hoetzl, Michael Maurer, Ilse Kranner, Sara Weiss, Frank Weihmann

**Affiliations:** 1 Institute of Zoology, University of Graz, Graz, Austria; 2 Institute for Computer Graphics and Vision, Graz University of Technology, Graz, Austria; 3 Institute of Botany, University of Innsbruck, Innsbruck, Austria; University of Arizona, United States of America

## Abstract

Shimmering is a defence behaviour in giant honeybees (*Apis dorsata*), whereby bees on the nest surface flip their abdomen upwards in a Mexican wave-like process. However, information spreads faster than can be ascribed to bucket bridging, which is the transfer of information from one individual to an adjacent one. We identified a saltatoric process that speeds up shimmering by the generation of daughter waves, which subsequently merge with the parental wave, producing a new wave front. Motion patterns of individual “focus” bees (n = 10,894) and their shimmering-active neighbours (n = 459,558) were measured with high-resolution video recording and stereoscopic imaging. Three types of shimmering-active surface bees were distinguished by their communication status, termed “agents”: “Bucket-bridging” agents comprised 74.98% of all agents, affected 88.17% of their neighbours, and transferred information at a velocity of v = 0.317±0.015 m/s. “Chain-tail” agents comprised 9.20% of the agents, were activated by 6.35% of their neighbours, but did not motivate others to participate in the wave. “Generator agents” comprised 15.82% of agents, showed abdominal flipping before the arrival of the main wave front, and initiated daughter waves. They affected 6.75% of their neighbourhood and speeded up the compound shimmering process compared to bucket bridging alone by 41.5% to v = 0.514±0.019 m/s. The main direction of shimmering was reinforced by 35.82% of agents, whereas the contribution of the complementing agents was fuzzy. We discuss that the saltatoric process could enable the bees to instantly recruit larger cohorts to participate in shimmering and to respond rapidly to changes in flight direction of preying wasps. A third, non-exclusive explanation is that at a distance of up to three metres from the nest the acceleration of shimmering could notably contribute to the startle response in mammals and birds.

## Introduction

**Figure 1:**
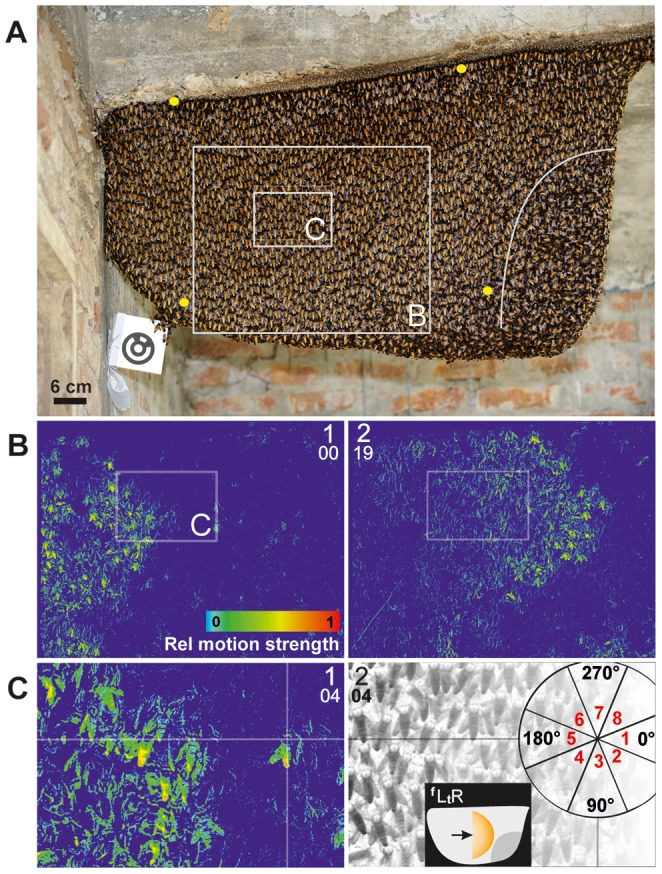
Experimental nest of Apis dorsata. (**A**) High resolution photo of *nest B*; grey open squares refer to the boundaries of the images of the panels B and C; the four yellow points mark the corners of the image part in Movies S1, S3 and S5. In the mouth zone the surface bees are randomly oriented (which is visible at the right side and below the white curve); peripheral to the mouth zone the surface bees are mostly vertically oriented, with their heads upwards and abdomens downwards. (B–C) Differential images (see image analysis in Methods, [7]) from high resolution video recordings (fps = 60 Hz) of nest part specified in the panel A (real-world measures of panels: B/43×34 cm; C/14×10 cm); large capital numbers show the panel count, small numbers give the relative count of frames during the propagation of the shimmering process which took place from left to right (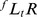) in the image. The rectangle in panel B indicates the boundaries of panel C. The time difference between the images of B_1_ and B_2_ was 317 ms [≡19 ff]. Pseudo colours (panels B_1,2_ and C_1_) visualize 8-bit 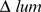 *v*alues of *motion strength* pixelwise in the rainbow palette with *blue* = 0, *red* = 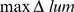 (see scale in B_1_ and Methods). (C_2_) The same image as in panel C_1_ but in black-and-white and inverted for better discrimination of the abdomens; at the crossing points of the both marker lines an *agent bee* was selected which was moving solitarily ahead the shimmering front which was proceeding from the left to right; the schematic on the right gives the angular sectors of the *near neighbourhood* of this *focus bee* (with the directional categories; 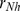
<40 mm).

Shimmering [1]–[9] in giant honeybees (*Apis dorsata*) is one of the most sophisticated communication behaviours in insects. Shimmering takes place at the nest surface [1], [3]–[9], which constitutes a matrix of densely clustered individuals arranged in a multi-layered stratum, forming the *bee curtain* [10] around a central, flat comb (Fig. 1A). In shimmering, individual bees flip their abdomens upwards, producing wave-like patterns (Movies S1, S2, S3, S4, S5, S6), which propagate across the nest surface in about one second, also affecting sub-surface layers [7]–[8]. Giant honeybees show this collective behaviour in response to threatening enemies, in particular to predatory wasps [6]. It is commonly accepted that colony defence is the primary goal of shimmering [6], [11]–[12].

Some of the mechanisms of shimmering have been clarified [6]–[9], [11]–[14], but it is not fully understood how the waves propagate [7]–[9]. Shimmering has been described as a Mexican-wave-like process [15], following the principles of *bucket-bridging* [7], [9] to transfer information along a chain of agents, such as passing a bucket of water from one person to another to extinguish a fire in the old days. The *bucket-bridging hypothesis* of shimmering [7], [9], [12] predicts that surface bees are affected one-by-one *continuously* and *linearly* along a propagation line according to three principles: the first principle is that propagation is *directed*, whereby a surface bee is stimulated to participate in shimmering by her neighbour bees in those angular sectors where shimmering cohorts show maximal activity [9]. The second principle of *bucket bridging* is that shimmering activity proceeds steadily from one agent to the next in a *linear* fashion [7], [9]. The third principle is that information is transmitted in parallel chains of agents (for a summary of abbreviations and definitions, see Glossary S1). Theoretically, if information generated at a certain spot propagates in parallel queues at exactly the same velocity, the frontline of the wave would represent a single row of shimmering bees. Any deviation from this parallel propagation will broaden the wave front. The directivity in propagation based on these three principles was addressed in the *directed-trigger* hypothesis of *bucket bridging* [9].

Although shimmering is a collective, synchronized and cascading behaviour, each agent is seemingly free to decide whether to participate in shimmering or not. Furthermore, agents that do participate flip their abdomens at variable strengths and angles of up to 120°. The starting point of shimmering depends on the position of the threatening cue [11]–[12], from where it spreads into all directions, similar to mechanical analogues of water or sound waves [16]–[17]. However, shimmering does have different ways to propagate [9], [11]–[12]. Apart from *bucket-bridging* [7]–[9], shimmering waves appear to “jump” from specific sites to others [11]–[13], typically in increments of ten to fifteen surface bees, in a *saltatoric* process.

Here, we investigated the contribution of both, *bucket-bridging* [9] and *saltatoric* processes, to shimmering. The main characteristics of wave propagation were determined on the single bee level regarding recruitment, velocity and directionality, and three functional types of information transfer were distinguished. We found that the *saltatoric* process is associated with the generation of subordinate or *daughter* waves. By merging of *daughter* and *parental* waves, shimmering waves are speeded up by a factor of two to five, likely reinforcing the anti-predatory impact of shimmering [6].

## Materials and Methods

### Experimental conditions

#### Site and recording

The shimmering behaviour of giant honeybees was studied under field conditions during two expeditions to Nepal. The recording setup (see below) was established at three sites on the campus of the Tribhuvan University in Rampur (February 2009: nest A) and at the border of the Chitwan National park (February 2009: nest B [Fig. 1A]; November 2010: nest C). Two synchronized cameras were used to record black-and-white images with a resolution of 2,352×1,728 pixels (px), whereby one pixel covered approximately 0.30 mm in real-world coordinates. Therefore, the characteristic abdomen width of 6 mm of a giant honeybee was imaged by roughly 20 px. The cameras captured 60 frames per s (fps), resolving the abdomen-flipping phase of an individual bee of 200 ms within 12 frames (for further details see [6]–[9], [12]–[13]). Nests were also filmed with a high-definition video camera (Panasonic HVX 200) at 50 fps and a resolution of 1,280×720 px from distances between 1.5–10 m, whereby the camera angle always covered the whole nest. Nest B was used for an automated in-depth analysis of the data recorded for approximately 11,000 focus bees and 460,000 neighbour bees (see below). The validity of the results from nest B was confirmed by manual analysis of selected aspects from other nests (see Results). This confirmation, together with our previous experience with many other nests [5]–[9], [11]–[13] made us confident that the parameters used to describe shimmering behaviour previously and in this paper do not represent colony- or nest-specific traits, but can be considered as representative for the behaviour of *Apis dorsata* generally.

#### Dummy wasp stimulation of bees

For eliciting shimmering waves, colonies were stimulated with a dummy wasp fixed to a cable car device [7]–[9], [13] at the sun-exposed side of the nests. A dummy with white, yellow and black stripes was made of Styrofoam (LxWxH: 40×15×15 mm) and suspended from a thin thread. Close to the mouth zone, the dummy was swung horizontally at an angle of 90° perpendicularly to the nest. The movement of the dummy was computer-controlled at variable velocities (0.1–0.5 m/s) and directions (towards and away from the nest). For more intense stimulation the dummy was connected to a 1.5 m long stick and moved manually (see Movie S1).

### Characterization of shimmering

#### Identification of agents

In a giant honeybee nest, the colony members are arranged on both sides of the central comb in several layers, whereby several functional regions can be discerned, such as the *mouth* zone [10], the attachment zone, the rim zone and the *quiescent* zone [6]–[9], [11]–[13]. Shimmering behaviour is mainly seen in surface bees in the *quiescent* zone ([7]–[9], [12]; Movies S1, S2, S3, S4, S5, S6), flipping their abdomens upwards at an angle of up to 120°. In each frame, such agents were identified individually by stereoscopic imaging [7], [9] using the coordinates of their thoraces, measured as x- (horizontal directions), y- (vertical directions) and z- (directions towards and away from the comb) positions at resolutions of fractions of a millimetre. More than 600 agents were continuously tracked in successive frames throughout multiple shimmering processes. A total of over 50 episodes of 2 min duration, each comprising 2 waves, were recorded and analysed under defined stimulation protocols [7], [9].

#### Definition of spreading directions

The bee curtain of giant honeybee nests displays a polar topology where the individual bees are arranged with their heads upwards and their abdomens downwards ([7], [9]; Fig. 1). Four key directions of wave spreading were defined (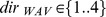), namely two horizontal directions (*from Left to Right* [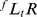] and *from Right to Left* [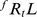], whereby *Left* and *Right* refer to the recorded image), and two vertical directions (*from Top to Bottom* [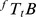] and *from Bottom to Top* [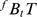]).

#### Assessment of motion strength

The image-analysis software Image-pro plus (Media Cybernetics) was used to measure the parameter 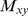 [7], [9] that quantifies the motion of an individual agent bee. 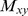 refers to the luminance changes (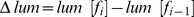) in a 60×60 px zone around the thorax of the selected agent bee and was assessed in differential images (e.g. Fig. 1B,C_1_; [6]–[9], [12]–[13], [15]) by pixel-wise subtractions of data from one frame (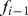) to the consecutive one (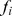) at intervals of 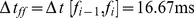 (fps = 60 Hz). The value 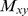 includes mainly positional changes in horizontal (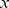-) and vertical (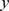-) directions, as well as movements of head, abdomen and extremities, such as legs, antennae or wings. Bees can move actively during *shimmering* [9]–[14], *flickering* [12]–[13] or locomotion (i.e., moving around on the nest surface or penetrating into or emerging from the subsurface layers of the nest). Bees can also be shifted passively when affected by the movements of their immediate neighbours [7]. Values for quiescent conditions were 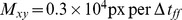, and for massive shimmering activity they were 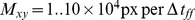. Values for 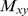 were normalised as 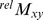 relative to the maxima in each recording [7], [9], because they depend on conditions of video recording such as lighting and zoom mode used, and on image analysis tools such as subtraction, filtering and segmentation.

#### Determination of time zero

**Figure 2:**
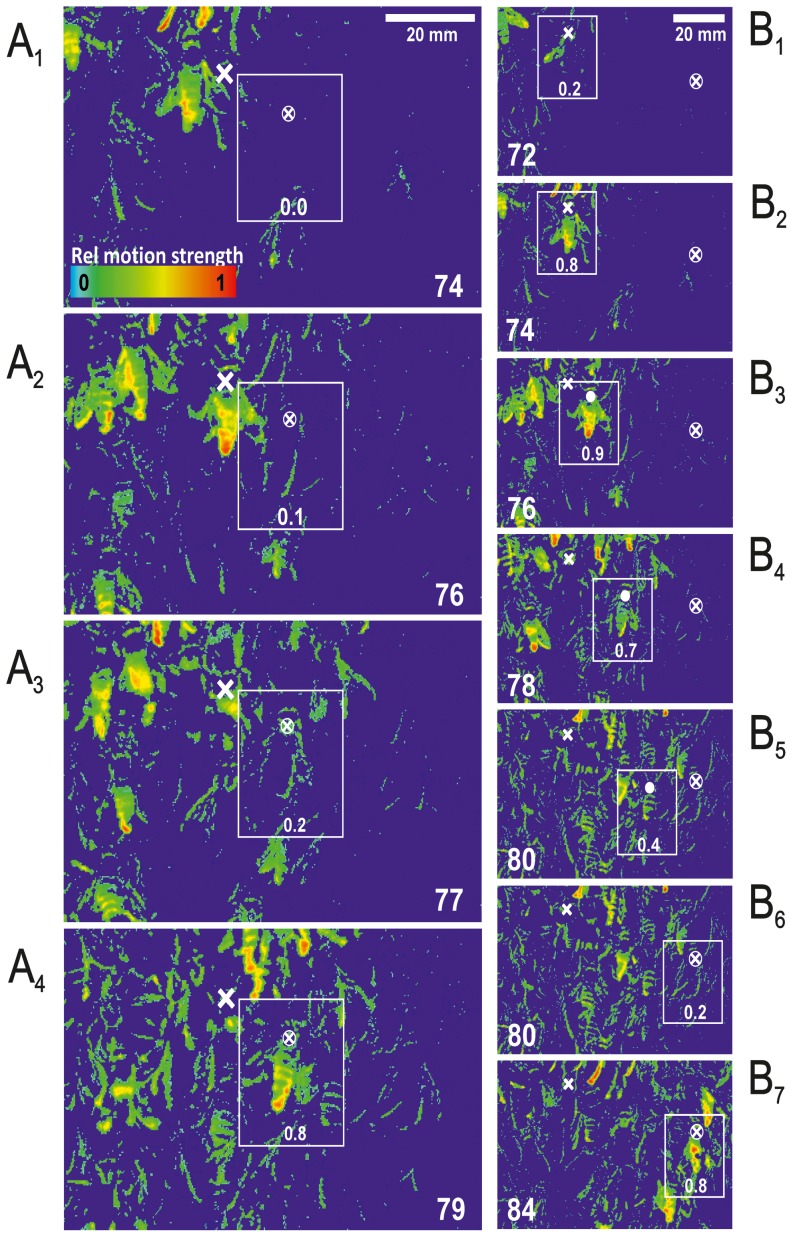
Two examples of bucket-bridging in the signal propagation of shimmering of A. dorsata. (A_1–4_) Information transfer from left to right (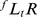) in the images, displayed over six frames (from f 74 to f 79≡100 ms) from the *focus bee* (whose thoracic position was marked with a white cross throughout the frames) to one of her neighbours (whose thoracic position was marked with a white cross in a white circle); differential images represent luminance [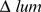] patterns according to the rainbow scale displayed in panel A_1_ (see Fig. 1 and Methods), the grey open rectangles signify the extension of the receiver agent, the numbers at the bottom of the rectangles refer to the relative activation level of the *shimmering incident* (whereby the pixel-by-pixel luminance differences were scaled on the rainbow palette with 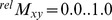; for the estimation of the momentary expression of motion strength, see Equations 2a,b for retrograde and prograde extrapolation in Methods under “Benchmarking *bucket-bridging*”). (B_1–7_) Information transfer from a *focus bee* (thoracic position marked with a white cross) over a chain of four neighbours to a target agent; the thoracic positions of intermediate neighbours were marked by closed white circles, that of the target agent with a white cross inserted in a white circle; this target bee was passively moved from B_4_–B_6_, and flipped her abdomen in B_7_); the white open rectangles signify the range of the respective agent along the selected chain, the numbers inside the rectangles give the respective activation levels.

**Figure 3:**
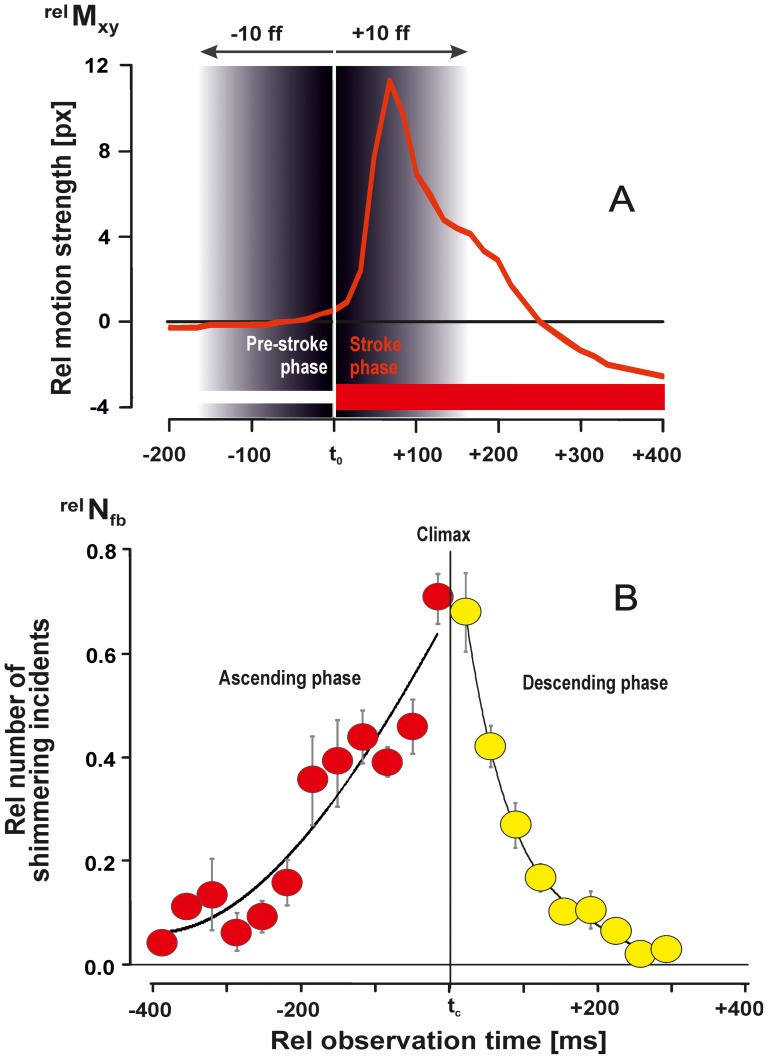
Definition of a shimmering incident and of the three phases of a shimmering wave. (A) Typical time course of the motion of an individual *focus bee* during abdominal flipping (termed *shimmering incident*): ordinate: relative motion strength assessed by 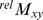 (for definitions, see Methods). Abscissa, observation time in ms; 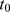, start time of the *shimmering incident* of the agent, and start time of the *stroke* phase, synonymous to the *post-[* 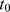 *]*-*stroke* phase; Grey shaded zone, the time window of ±167 ms (≡
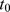
±10 ff) for categorizing the communication *status* of agents (see text). (B) The three phases (*ascending*, *climax*, *descending*) of a shimmering wave can be categorized by the course of recruitment of agents participating in a wave, quantified by the relative number of *shimmering incidents* which were traced per image at the relative observation time 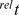 with 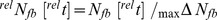 [per wave] and 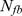 = 10,894 (see Methods; n = 25 episodes of 2 min duration). Full circles, arithmetical, vertical bars, SEMs; n = 40 waves. The time 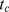 gives the time point of the *climax* when the maximum number of *shimmering incidents* (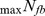) occurred simultaneously. Red colour marks the *ascending* phase (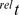
<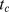) of the shimmering waves, yellow colour marks the *descending* phase (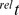
>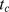).

When the wave front arrived at an agent bee, weak deflections in 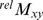 values of the agent bee were assessed [7], [9] even before she started to flip her abdomen (Fig. 2). Such small motions of an agent bee were caused by the shimmering-active neighbours. During abdominal flipping, the 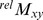 value sharply rises and peaks within 40 ms. In addition, the arrival of the wave can be traced by this parameter some frames before the abdominal flipping, which caused a slight increase in the 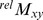 value (for details, see Figs. 2,3A and [7]). This transient increase allowed identifying a *shimmering incident* defined as the flipping of the abdomen of an individual agent bee participating in the shimmering wave. A *shimmering incident* was only considered if the agent exceeded the threshold (th) value of 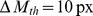 for at least five successive frames in the differential images (Equation 1). This criterion served to supress noise.
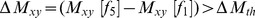
(1)


For each *shimmering incident* (Figs. 2,3A) the following parameters were assessed: (a) the start time zero (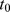) was defined one frame before the sharp rise of 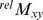 detected by the trigger algorithm at the time 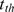 when the motion strength exceeded the threshold value according to 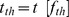 and 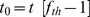.

**Figure 4:**
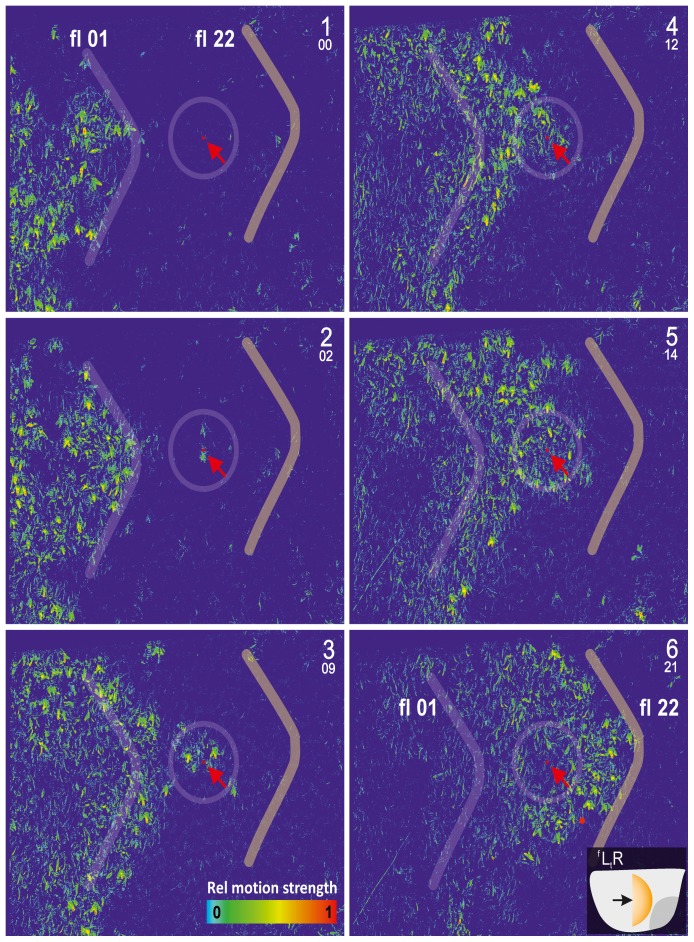
Propagation of a shimmering wave across the nest surface (survey view). This example refers to a wave that spread from left to right (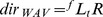) in the image. Capital letters in the right corners (1–6) show the panel count, small numbers give the relative count of selected frames (ff 01–22), comparable to Figs. 1, 2 and 4 (for distance measures, see Fig. 1B). The pseudo colour bar in panel 3 shows the scale for the relative motion strength (see Fig. 2). For better comparison, the thick grey line fl 01 gives the *front line* of the (*parental*) wave manually drawn in panel 1 (at f 01), and the line fl 22 shows the front line of the same wave 384 ms later (at ff 22); red points marked with red arrows show the thorax of the *focus bee* selected in this wave, which flipped her abdomen at f 02 and generated a *daughter* wave that subsequently merged with the *parental* wave at f 12. The circles around the *focus bee* indicate the range of the *near neighbourhood* (<40 mm).

**Figure 5:**
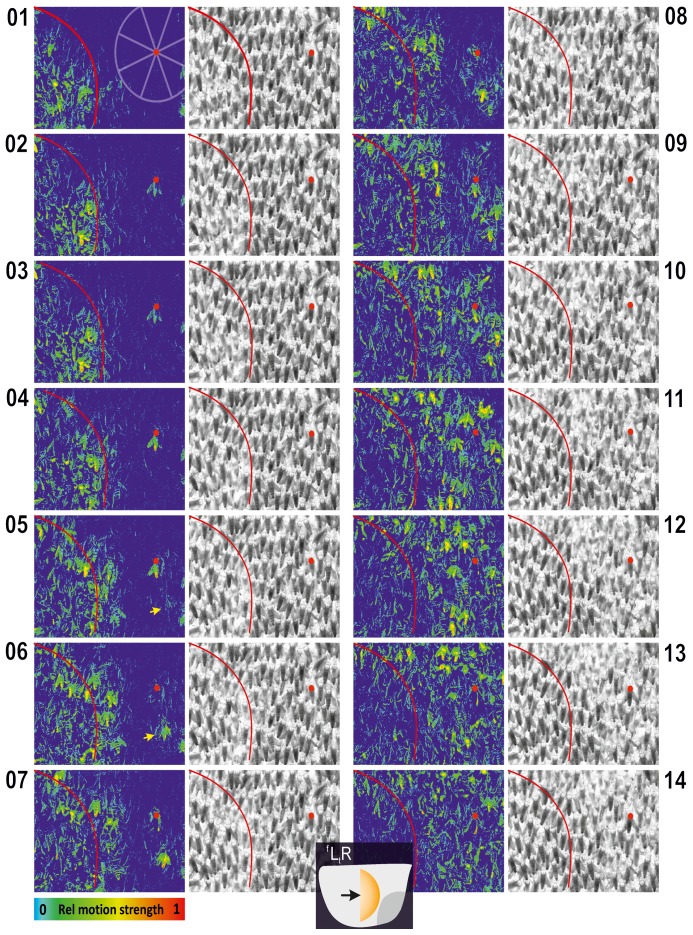
Propagation of a shimmering wave across the nest surface (detail view). The same wave is shown as in Fig. 3 but zoomed (for distance measures, see Fig. 1C). Continuous sequence of frames as pseudo coloured differential images and inverted video images; numbers (01–14) indicate the relative frame counts (fps = 60 Hz with interframe intervals of 16.67 ms). Scale below panel f 07 shows the relative motion strength (see Fig. 2). Red dots indicate the selected *focus bee* (the same as in Fig. 4) that started to flip her abdomen at f 02, acting as a *generator bee* for the subsequent *daughter* wave. One of her neighbours (marked with a yellow arrow) was passively shifted in f 05 and started the abdominal flip in f 06. The resulting *daughter* wave merged with the *parental* wave at f 10. The red curves on the left side of the images are shown for comparison with the initial position of the *parental* wave front as indicated in f 01.

(b) The motion strength of the *shimmering incident* of a selected agent was defined by the maximal 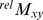 value measured within 10 frames after 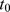. These strength values were graded in eight steps proportional to the 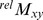 values (with motion strength categories 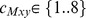). Hereby, the assignment of 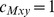 was particularly important, as this minimum level of active shimmering motion of a selected agent bee had to be clearly distinguished from unambiguous (passive) sub-threshold motions (cf. Fig. 2A_2_). Only active, supra-threshold movements with 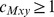 were considered. In contrast to the agent-related 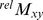 values the pseudo colours of differential images (Figs. 1–2,4–5) visualize the relative motion strength by the luminance differences pixel by pixel, scaled with 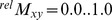 on the rainbow palette.

#### Focus bees and their neighbours

The participation in shimmering greatly varies from agent to agent and ranges from passive motions (Fig. 2A_2_) caused by the approaching wave front to active participation with abdominal flipping at variable strengths (Fig. 3A). To support the automated evaluation and sorting of the various types of *shimmering incidents*, the concept of the *focus bee* was introduced. For every *focus bee*, two sets of *neighbour bees* (nb) were defined, which were positioned in the *near neighbourhood* (<40 mm; Fig. 1C_2_) and in the *far neighbourhood* (>40 mm, <100 mm). The numbers were distinguished according to whether these neighbour agents had participated in shimmering before (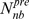) the wave front arrived at the *focus bee*, or afterwards (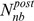). Thus, an agent bee was treated either as a *focus bee*, or as a *neighbour* bee.

In the in-depth study, *focus-bee* or *neighbour-bee* status was assigned to agents regarding all four main wave directions (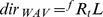: 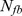 = 2,823; 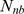 = 138,190 [for >40 mm, <100 mm]/29,248 [for <40 mm]; 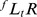: 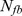 = 3,098; 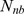 = 159,417/44,939; 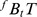: 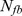 = 3,396; 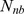 = 105,356/13,211; 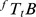: 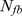 = 1,577; 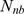 = 56,595/9,539; all wave directions: 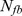 = 10,894; 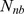 = 459,558/96,937, where fb and nb stand for *focus bee* and *neighbour bee*, respectively). For most results shown in this paper, waves in the direction 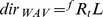 were taken as representative of all four data sets.

#### Trigger neighbours

The time point 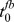 of a *shimmering incident* of a *focus bee* was used to define the potential *trigger neighbour bee* for a given *shimmering incident* (Fig. 3) by considering two criteria: First, the *trigger neighbour* must have already participated in the same wave before the *focus bee*; however, this participation was only considered if this neighbour was active within the time window of 88 ms (−5 ff) prior to 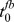. Second, the agent defined as *trigger neighbour* had to be positioned closest of all other candidates to the *focus bee* and in her *near neighbourhood*. This criterion excluded those bees from analysis that flipped their abdomen outside the *near neighbourhood* (in many cases such bees generated *daughter* waves; Figs. 1–2, 4–5). The *trigger direction* of a *focus bee* (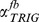) was defined by the angle of the direction of her trigger neighbour, measured from the position of the *focus bee*, categorized according to the eight sectors of neighbourhood (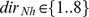; Fig. 1C_2_) in which the trigger neighbour was positioned.

#### Relative time scales of focus bees and their neighbours

The time courses of the *shimmering incidents* of selected neighbour bees were synchronized to the time 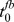. This synchronization conjoined the movements of *focus bees* and her triggering neighbours with the positional (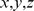) parameters and the 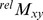 values. This enabled sorting and pooling of identified *focus bees* and their neighbours, collected at different locations and times according to *main wave directions* (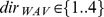), *motion strengths* (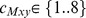), and *trigger directions* (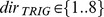) for further statistical analysis.

#### Benchmarking of bucket-bridging

*Bucket bridging* was characterised for manually selected agents by the assessment of the information transfer between two neighbouring bees (Fig. 2A), and over a chain of adjacent agents (Fig. 2B). The thorax-to-thorax distances were calculated in mm. A motion detection method was used that utilized differential images (see above) considering the following criteria [9]: (a) Chains of agent pairs were selected (Fig. 2), in which *emitter bees* (which flipped their abdomen first) could clearly be distinguished from *receiver bees* (which followed the action of the *emitter* bee) throughout a continuous and linear sequence of actions. (b) *Emitters* and *receivers* participated in the same *chain* (see Introduction and [9]). (c) Bees were excluded from data assessment if they did not show any interaction, if they were activated synchronously (instead of sequentially, i.e. they did not participate in the same chain) and if they showed abdominal flipping with a delay larger than 167 ms (≡10 ff). (d) In differential images, the temporal information transfer was estimated using the abdominal flipping of the *emitter* and the *receiver* bee. The time 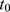 was determined by weighting 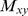 values in deciles whereby 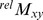 = 0.0 indicated *quiescence* of the *focus bee* (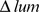
<8), and 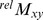 = 1.0 defined a fully lifted abdomen. The first and last frames (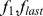) with traces of motions of individual agents were weighted accordingly (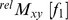, 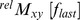) to estimate the start and end points of information transfer (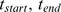) by bucket bridging. This measure retraced the start point of abdominal flipping by retrograde extrapolation (Equation 2a) on the basis of the weighting value 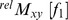. Similarly, the abdominal flipping of the last bee in a chain was calculated by determining the last frame (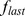) with motion activity by prograde extrapolation (Equation 2b).
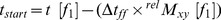
(2a)

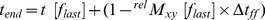
(2b)


### Ethics Statement

The research expedition to Chitwan, Nepal, entitled “Study on the behaviour of the giant honeybees: Observations and recording of behaviours at the nesting site” was supported by the Rector of the Centre for International Relations of the Tribhuvan University (Kathmandu, Nepal).

## Results

### Bucket-bridging

An example of *bucket bridging* is shown in differential images in Fig. 2A_1–4_ (ff 74–79 corresponding to 100 ms). The *emitter bee* showed maximum activity in f 76. The silhouette of the *receiver bee* was already visible in f 76, when it was slightly moved passively by the advancing wave front. In f 77, the shape of the right forewing of the *receiver bee* appeared, indicating active participation in shimmering. In f 79 her abdomen started to be lifted actively, followed by a massive motion of the whole body accompanied by a beat of both wings.

An individual abdominal flipping typically lasts 67.16±0.97 ms (mean ± SE; n = 174 abdominal flips; Figs. 2, 3A, 4; [14]). The manually evaluated shimmering waves (n = 47) showed that information transfer between adjacent shimmering-active neighbours (positioned at distances of 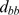 15.94±0.66 mm, where bb stands for *bucket bridging*) was completed within 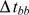 = 39.16±2.84 ms (Fig. 2A_1–4_), corresponding to a velocity of 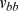 = 0.5085±0.0413 m/s. However, with 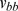 = 0.317±0.0145 m/s the speed was lower when assessed over a short chain of agents (Fig. 2B, 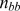 = 2.60±0.17 bees; 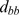 = 43.32±1.98 mm; 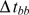 = 107.90±8.58 ms; n = 40 shimmering waves). We then used this speed value (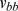 = 0.317 for nest B) as a benchmark for characterizing the *bucket-bridging* process. For comparison, similar speed values were found for nests A and C (nest A: 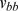 = 0.2461±0.0193 m/s, n = 28 shimmering waves; nest C: 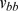 = 0.3598±0.0263 m/s, n = 26).

### Saltatoric processes

*Saltatoric* wave propagation involves bees that are more than 80 mm away from the approaching wave front, and lift their abdomens typically 30–50 ms earlier than other participants in their own *near neighbourhood*. These bees generate *daughter* waves and are termed *generator* agents. In Figs. 4,5 (compare Movies S1, S2, S3, S4, S5, S6), one of these *generator* agents is marked by a red point on the thorax. During the abdominal lifting of this agent (ff 02–06, Fig. 5), the *parental* wave (pw) moved from left to right in the image. Using the position of the wave front in two subsequent frames the velocity of the *parental* wave was found to be 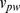 = 0.239 m/s, which is in the same order of magnitude as calculated for *bucket bridging* in the same nest (Fig. 2A).

In f 09 of Fig. 4 the *daughter* wave is visible as a small circular batch, and in f 12 this *daughter* wave started to merge with the *parental* wave. The frontline of the *parental* wave “jumped” from the left to the right side of the *daughter* wave (from f 10, immediately before the *daughter* wave merged with the *parental* wave, to f 11, just after merging) within 16.67 ms or even less. The frontline of the merged waves advanced over a distance of 160 mm, which corresponds to an acceleration of the shimmering velocity (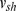) to a value of at least 0.960 m/s by *saltatoric* information transfer. This is roughly three times faster than *bucket-bridging* alone.

Fig. 5 demonstrates the *saltatoric* information transfer in more detail for the same *focus bee*. This bee started abdominal flipping in f 02, and one of her neighbours joined 70 ms later (marked by a yellow arrow, first visible in ff 04–05). Subsequently, other neighbours followed, producing a *daughter* wave. The *parental* wave on the left side of the image continued to spread to the right, while the daughter wave spread into all directions by *bucket bridging*. Finally, 170 ms after the start of abdominal flipping of the *generator* bee (in f 10), the *daughter* wave merged on its left side with the *parental* wave, while the frontline of the merged waves “jumped” to the right side of the former *daughter* wave.

### Propagation velocity of shimmering waves

A shimmering wave lasts up to 800 ms (Fig. 3B) and includes the *ascending* phase of 200–300 ms, in which the number of synchronously shimmering surface bees increases to the maximum, the *climax* phase, when a maximum number of agents were simultaneously active (lasting approximately 200 ms), and the *descending* phase (300–400 ms), in which the number of synchronously shimmering surface bees decreases ([6], [12] and Fig. 3B). When measured from the start to the end of a wave by the detection of the positions of the wave fronts in differential images (Figs. 1–2,4–5), 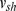 was calculated to be 0.367±0.020 m/s (n = 10 waves, see Movies S1, S2, S3), similarly slow as found for *bucket bridging*.

Alternatively, when measured during the *climax* phase, 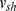 was 0.514±0.019 m/s, significantly (P<0.01, Student's test) faster than the benchmark value for *bucket bridging* (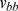 = 0.325 m/s). The 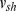 value for the *climax* phase was calculated for the wave direction 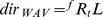 regarding nest B by selecting pairs of agents that were positioned horizontally (at an angle of 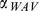 = 7.95±5.40°; n = 41 waves; definition of angle of wave direction in Fig. 1C_2_) at a distance of 112.90±4.08 mm between those *emitters* and *receivers* that were subsequently affected by the same wave fronts within ten frames or more (226.06±8.21 ms) in a straight line (cf. Fig. 2B).

This 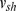 value of 0.514±0.019 was taken as a benchmark value for the mixed propagation mode (*bucket bridging* and *saltatoric* processing). Hence, 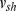 during the *climax* phase is faster than during the start or the end phases, likely due to the higher probability of the occurrence of *saltatoric* events.

### Communication statuses of agents

**Figure 6:**
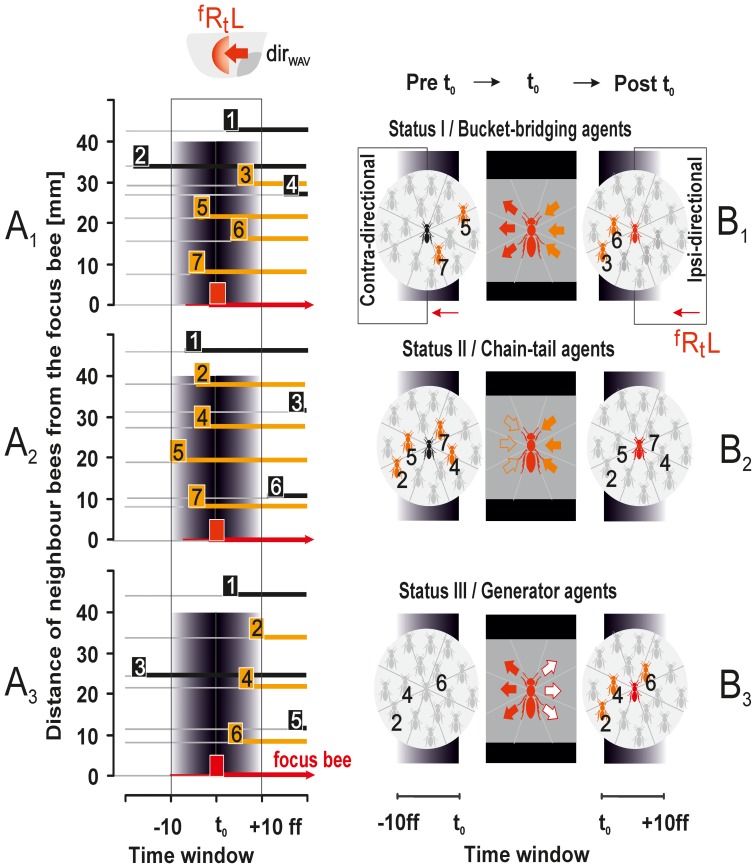
Definition of three communication statuses of surface bees (shown for the wave direction 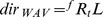
 **).** Sketches of activity plots for distinguishing the *statuses* (*I,II,III*) of *focus bees*. (A_1–3_) *Focus bees* are marked by red full rectangles at 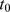; abscissa, time in frames (fps = 60 Hz) in relation to [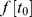] when the *focus bee* started her flipping; ordinate, distance of neighbours from the *focus bee* in mm; grey thin horizontal lines symbolize the *quiescent* state of the sample agents; small full rectangles with numbers mark the onset of their abdominal flips; orange coding refers to positions of the neighbours within the *near neighbourhood* (<40 mm) with flipping activities within the critical time window of ±10 ff; black coding refers to complimentary attributes (>40 mm; >|±10 ff|). Thick horizontal lines (black, orange, red) symbolize that abdomen flipping is still going on. (B_1–3_) The panels *Pre t_0_* and *Post t_0_* display the *near neighbourhood* of a *focus bee* which is marked black when quiescent and marked red when she flips her abdomen. The numbers and colour codes of shimmering-active neighbours are the same as in the panels A_1–3_; arrows of the central panels (regarding the time 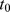) explain the directions of information transfer: *status I* (*bucket-bridging*) agents in the *pre*-*stroke* (*Pre t_0_*) phase receive mechanical information (full orange arrows) predominantly from the side from where the wave came, in the *post*-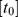-*stroke* (*Post t_0_*) phase information is transmitted predominantly to the side where the wave is spreading to (open red arrows); s*tatus II* (*chain-tail*) agents receive and emit mechanical information but fail to recruit other neighbours as transmitters; *status III* (*generator*) agents utilize predominantly visual information from the threatening cues, but not from shimmering-active agents in their *near neighbourhood*. They are leaders in emitting mechanical information and generate *parental* or *daughter* waves.

The contributions of individual agents to the spreading of shimmering waves, i.e. to *bucket bridging* or *saltatoric* propagation were assessed by automated techniques using the algorithms described below. The “communication status” of a f*ocus bee* was characterized spatially and temporally by considering her *neighbours* in the *near neighbourhood* as a reference region (<40 mm, see schematic in Fig. 6), and the *pre-stroke* and *post-stroke* intervals of ±167 ms (≡±10 ff) as a reference time window, whereby the abdomen flipping commenced at the time 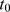. Shimmering waves were assessed regarding four wave directions (Table S1, [7]), and for in-depth analysis of communication status the wave direction 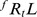 (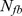 = 4,025; 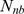 = 29,248 [for <40 mm]) was selected as representative for all waves.

**Figure 7:**
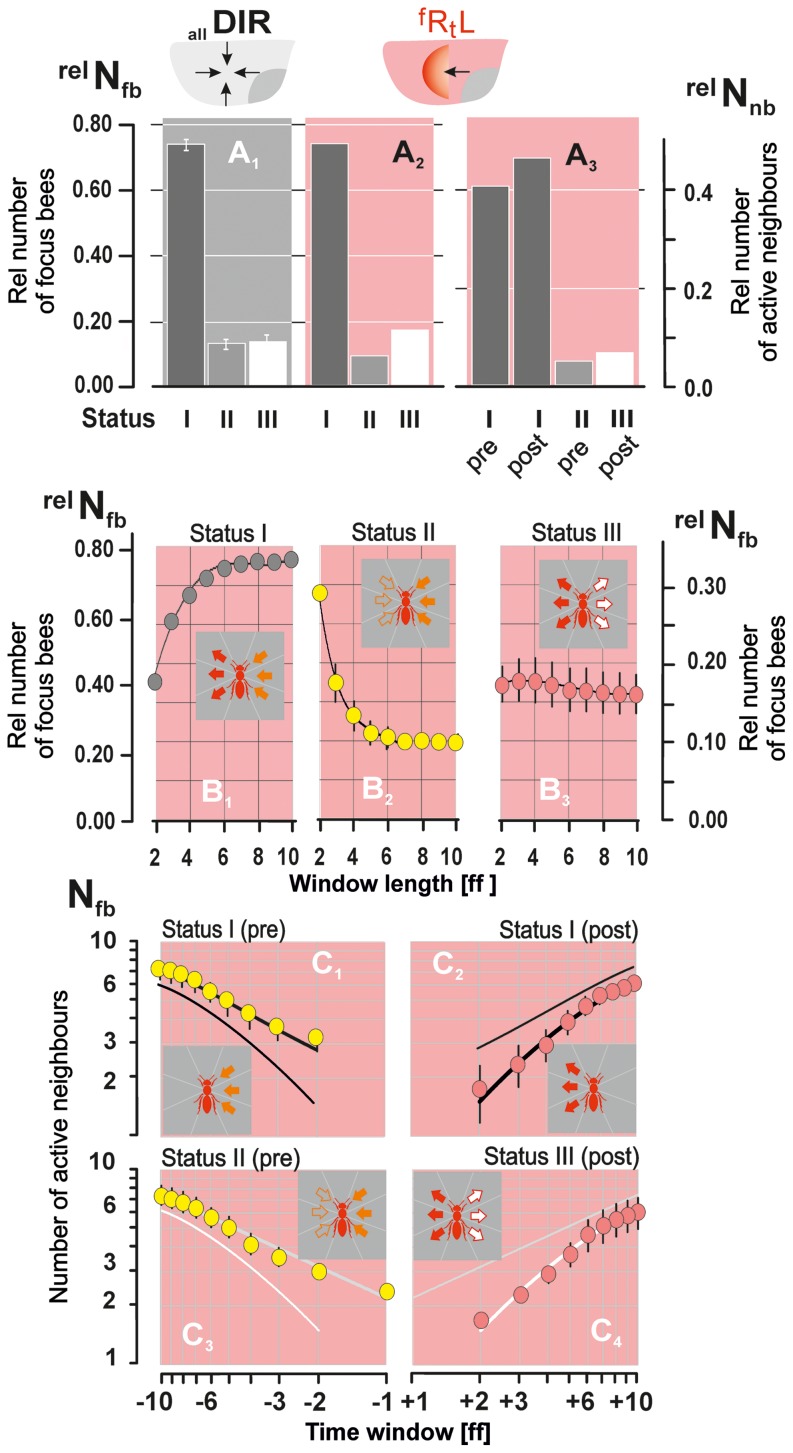
Properties of status I–III agents. (A_1–3_) Rates of *status I–III* agents (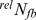) regarding the four directions of wave propagation 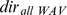 (grey background, panel A_1_, 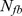 = 13,678) and regarding the wave direction 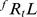 (pink background, panel A_2_, 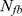 = 4,025); panel A_3_, rates of shimmering-active neighbours (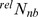) of *status I–III* agents (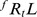, 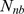 = 29,248) in the respective *pre-stroke* (pre) and *post-stroke* (post) phases; see also Table S1. (B_1–3_) Rates of *status I–III* agents (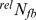) in dependence of the length of the discrimination window (abscissa in [ff], fps = 60 Hz). (C_1–4_) Numbers of shimmering-active neighbours (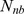) of *status I–III* agents depending on the length of the discrimination window (abscissa scaled as 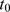
±ff); ff<0 refers to *pre-stroke* phase (yellow coding), ff>0 to *post*-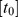-*stroke* phase (red coding). Columns (A) and full circles (B–C), arithmetical means; vertical bars, SEMs.

Three communication *statuses* (*I–III*) of *focus bees* were distinguished. *Status I agents* are driven by shimmering-active neighbours in the *pre-stroke* phase, positioned at their *ipsi-directional* side (from where the wave came), passing information on to their *contra-directional* side (to where the wave is continuing) in the *post-stroke* phase (Fig. 6A_1_,B_1_). This strategy of propagating information conforms to *bucket-bridging* [7], [9], and these bees are termed *bucket-bridging* agents. They comprised the majority of surface bees and were represented by 54.33% of agents at a reference window of ±2 ff (whereby “−2 ff” defines the time window in the *pre-stroke* phase and “+2 ff” the time window in the *post-stroke* phase). The number of *bucket-bridging* agents increased (Fig. 7A_1–2_,B_1_) with the reference time window (±10 ff) up to a value of 74.98±2.83%. This increase was due to the definition of *status II* agents (Fig. 6A_2_,B_2_: see below), which may turn into *bucket-bridging* agents with increasing length of the reference window (Fig. 7). The neighbours of the *bucket-bridging* agents were similarly large in number in the *pre-stroke* and *post-stroke* phases (Fig. 7C_1_, Table S2: 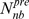 [at −10 ff] = 7.56±0.90; Fig. 7C_2_: 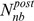 [at +10 ff] = 6.06±1.16) corresponding to 47.16% of all shimmering-active neighbours evaluated in the *pre*-*stroke* phase and to 41.01% of neighbours in the *post-stroke* phase.

**Figure 8:**
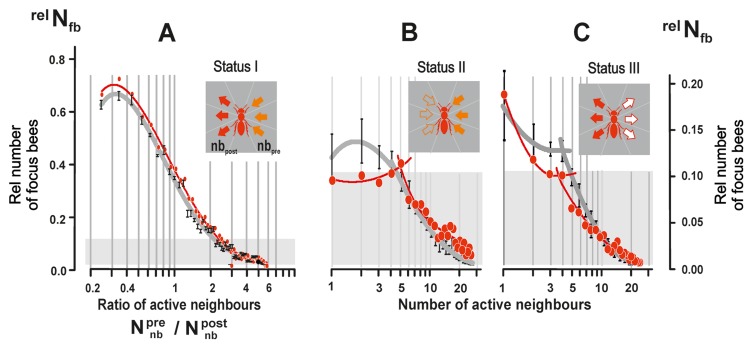
Relationship between status I–III agents and their neighbours. (A) The number of *status I* agents in dependence of the ratio of their shimmering–active neighbours in the *pre-stroke* and *post-stroke* phase (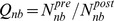); abscissa, 57 classes of 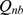 in steps of 0.1; ordinate, rate of *focus bees* whereby the value 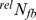 = 1.0 refers to the maximum number of cases per data set; red symbols: 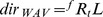 (n = 7 data sets); black symbols: 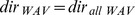 (n = 25 data sets); for regressions, see Table S1. (B,C) The rate of *focus bees* (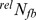) of *status II* & *III* in dependence of the numbers of their shimmering-active neighbours (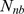); regressions of the mean values are shown into two parts: panel B: 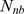
<5; 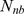
>5; panel C: 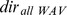: 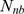
<6, 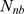
>5; 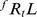: 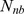
<5, 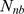
>4); for regressions, see Table S1. Full circles and mid positions of vertical bars, arithmetical means; vertical bars, SEMs.

In Fig. 8A, these *bucket-bridging* agents were sorted into two classes based on the number of shimmering-active neighbours in the *pre-stroke* and *post-stroke* phases (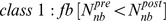); 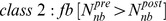). Hereby, the maximal numbers of neighbours found for *focus bees* were similar for both phases (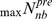 = 24; 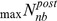 = 26) and both classes showed similar percentages (class 1: 50.27%; class 2: 49.73%; P = 0.1925, χ^2^ test) with a polynomial distribution, when sorted in steps of 0.1 parts of the ratio 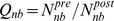; Table S1). This data symmetry applies to all phases of a shimmering wave (*ascending*, *climax* or *descending* phase; Fig. 3B and [6]). Furthermore, the selected wave direction 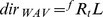 (red symbols in Fig. 8) was found to be representative of all four wave directions investigated (black symbols in Fig. 8), which shows that shimmering is invariant regarding the direction of wave propagation (cf. [9]).

S*tatus II agents* flip their abdomens triggered by their shimmering neighbours, (Fig. 6A_2_,B_2_), but thereafter their *near neighbourhood* becomes quiescent. These bees terminate the information transfer in their chains and are, henceforth termed *chain-tail* agents. The relative numbers of *chain-tail* agents increased from 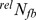 = 9.20±0.94% (at −10 ff, corresponding to 166.67 ms, where 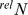 stands for relative number in per cent) to 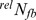 = 28.46±3.14% (at −2 ff≡33.33 ms; Fig. 7B_2_) and had 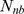 = 7.28±0.57 shimmering-active neighbours at −10 ff (Fig. 7C_3_, Table S1).

In contrast to *chain-tail* agents, *Status III agents* (Fig. 6A_3_,B_3_) are not triggered by their neighbours [<40 mm] in a reference time window in the *pre-stroke* phase of −10 ff, but flip their abdomens before their neighbours do so. They generate *parental* or *daughter* waves (as demonstrated in Figs. 1,4–5 and Movies S1, S2, S3, S4, S5, S6) and are termed *generator* agents. In contrast to *status-I* and *-II* agents, *generator* agents occurred at numbers which were roughly independent of the length of the *post-stroke* phase (at *+*3 ff≡50 ms: 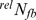 = 17.65±2.69%; at *+*10 ff: 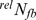 = 15.82±2.61%; Fig. 7B_3_, Table S1) and activated 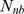 = 6.36±0.45 neighbours at +10 ff (Fig. 7C_4_; Table S1), which corresponds to 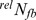 = 6.75% of the shimmering-active neighbours of all surface bees.

In Fig. 8B,C *chain-tail* and *generator* agents were sorted according to the number of shimmering-active neighbours in the *pre*-*stroke* (−10 ff) and *post-stroke* (+10 ff) phases. These data distributions allow discerning agents with less than five shimmering-active neighbours (subgroup 1), and those agents with more than five active neighbours (subgroup 2). In both agent types, subgroup 1 was larger, while subgroup 2 converged to zero with increasing numbers of active neighbours (Table S1). Hence, only few shimmering-active neighbours (<5) suffice to trigger both agent types.

### Acceleration of shimmering by saltatoric propagation

**Figure 9:**
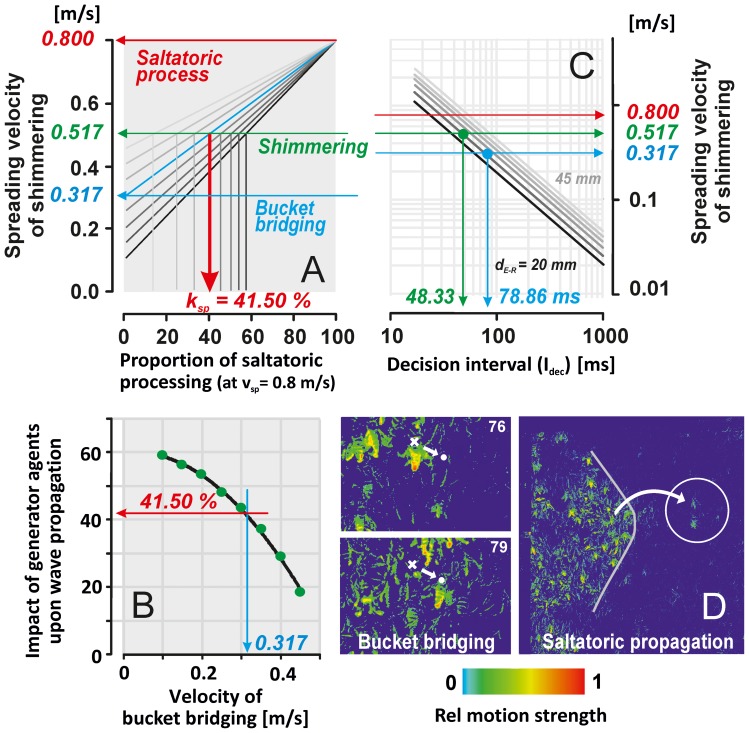
Mathematical model of the velocities in mixed-strategy wave propagation. (A) Lookup table for estimating the effect of the *saltatoric* process (sp) in shimmering in a mixed strategy with *bucket bridging* (bb): the proportion 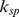 (abscissa) gives the impact of the *saltatoric* process on shimmering velocity (see Equations 3a,b) with 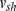 as ordinate and with 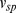 ( = 0.8 m/s) and 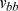 ( = var [0.10–0.45 m/s]) as parameters. For the benchmark values (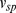 = 0.8 m/s; 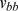 = 0.317 m/s; 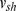 = 0.517 m/s) the impact of the *saltatoric* process on shimmering velocity is calculated as 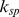 = 41.50%. (B) Ordinate, the impact of the *saltatoric* process on the empirical value of shimmering velocity (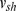 = 0.517 m/s) in dependence of *bucket bridging* (abscissa: 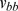) with the regression function: 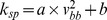 (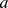 = −97.57, 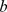 = 61.04; 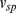 = 0.8 m/s). (C) Lookup table to estimate the *decision interval* (abscissa: 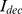 [ms]) of an agent bee in which she can decide to join a shimmering wave or not. 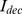 is calculated as the time interval in which a wave front spreads from an emitter (E) bee to a receiver (R) bee; ordinate, shimmering velocity 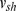 in m/s; the parameter 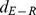 gives the distance between a *focus bee* and her adjacent neighbour (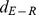 = 20 to 45 mm). For a typical distance (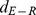 = 25 mm) the bee would have a time interval of 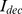 = 48.33 ms to “decide” to join the wave (for 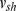 = 0.517 m/s, green arrows); for the benchmark value of *bucket bridging* (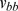 = 0.317 m/s, blue arrows) this time interval would be longer (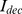 = 78.86 ms). (D) Pseudo coloured differential images (rel motion strength; see Figs. 1–2,4–5) to explain the principle of *bucket bridging* and of the *saltatoric* process.

The propagation of shimmering is based on complex synchronous and cascaded recruitment of surface bees, and was simplified in a mathematical model (see lookup tables in Fig. 9) that considers both the *bucket bridging* and the *saltatoric* propagation mode under climax conditions. This model allows to assess the impact of both processes on the 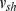 of the compound wave considering three parameters (where bb stands for *bucket bridging* and sp for the *saltatoric* propagation): (a) 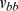, this is the 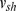 under solely *bucket-bridging* conditions (Fig. 9A), where 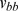 was varied in the model from 0.10 to 0.45 m/s (the benchmark value is 0.317 m/s, see above); (b) 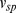, this is the 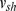 characterised by the *saltatoric* propagation, which was set to 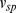 = 0.800 m/s (Fig. 9A–C); this velocity was slightly below the value of 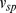 = 0.960 m/s that was found for a single *saltatoric* jolt of the wave front in the example in Fig. 4 (note that 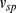 is only of theoretical relevance because a real shimmering wave never spreads saltatorically alone and that *bucket bridging* is the dominant propagation mode of shimmering); (c) the *distance* 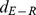 between the emitters and the receivers (E−R) in a chain of *bucket-bridging* agents; according to the conditions in the nest 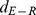 was varied from 20 to 45 mm (Fig. 9C,D). These three parameters were used to describe two wave properties that result from the combination of *bucket-bridging* and *saltatoric* propagation: First, the factor 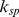 (Equations 3a,b) by which shimmering is speeded up by the saltatoric process (Fig. 9A,B).
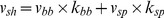
(3a)

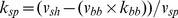
(3b)with the weighting factors in %: 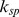 = 1..100 and 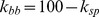.

For the above benchmark values under climax conditions (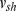 = 0.514 m/s; 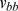 = 0.317 m/s) the impact of the saltatoric component (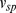 = 0.800 m/s) was 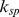 = 41.5% for 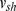 (Equation 3b) and the complementary 58.5% for 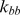 (Fig. 9B). Second, 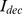 was considered, the time interval within which an agent “decides” whether or not to participate in shimmering (Fig. 9C; conforming with 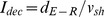).

The values of 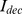 calculated for the distance of 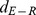 = 25 mm (which is characteristic for the side-to-side distance between bees at the surface of the bee curtain) ranged from 78.86 ms for mere *bucket-bridging* (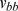 = 0.317 m/s) to 48.33 ms (Fig. 9C) for the combination of *bucket bridging* and *saltatoric* propagation Fig. 9A (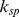 = 41.5%; 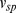 = 0.8 m/s).

### Directional control in shimmering

**Figure 10:**
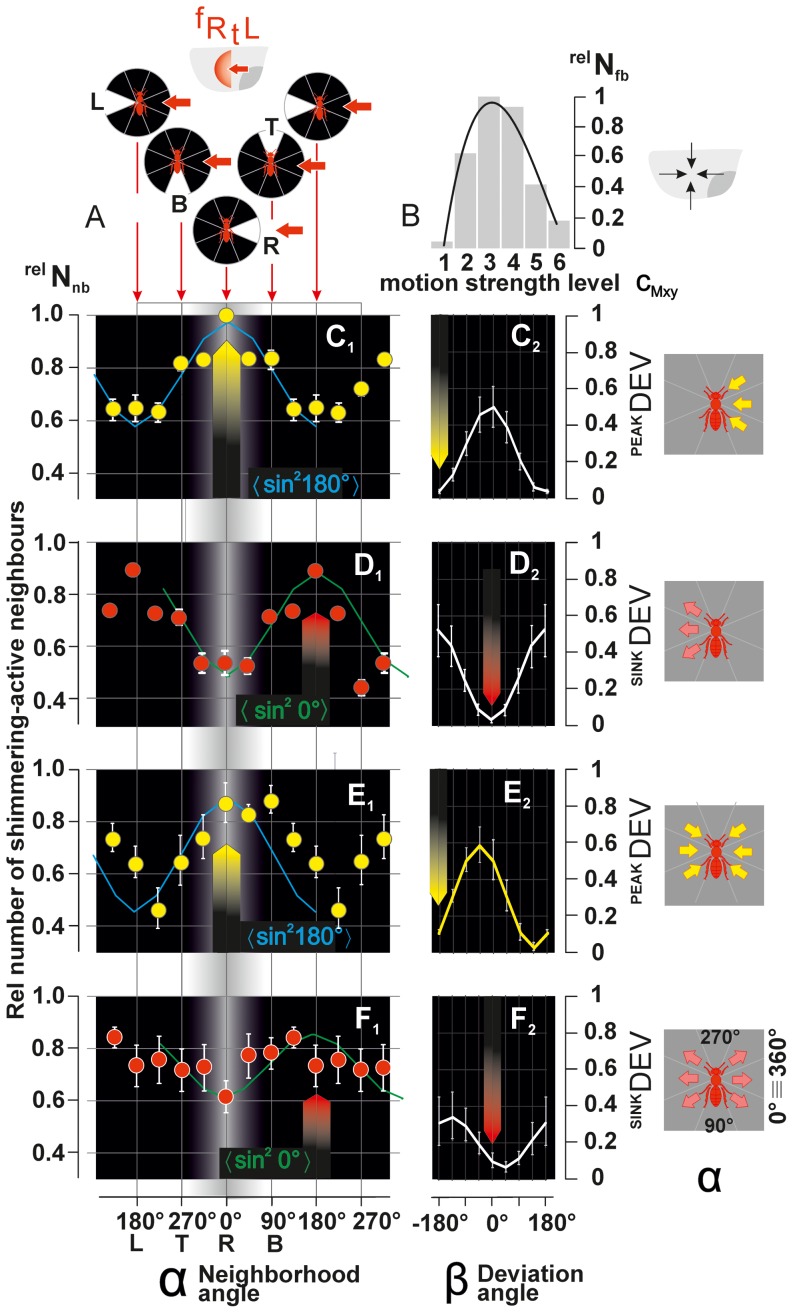
Directionality of status I–III agents. (A) Definitions: black circles indicate the *near neighbourhood* (<40 mm) with marked red *focus bees* in the centre; white angular sectors show the angles of neighbourhood (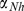, cf. Fig. 1C_2_) as indicated by thin red vertical arrows and the corresponding abscissa of the panels C_1_–F_1_ regarding the directions *right* [R: 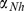 = 0°], *bottom* [B: 90°], *left* [L: 180°] and *top* [T: 270°]; thick red horizontal arrows indicate the direction of wave propagation, exemplified here for 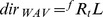; (B) Histogram of *focus bees* (*status I–III*) of all wave directions (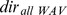: 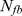 = 13,678) in dependence of levels of motion strength (abscissa: 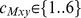; for statistics, see Table S1). (C_1_–F_1_) Relative numbers of shimmering-active neighbours (ordinate: 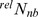 with 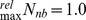 per communication status) of *focus bees* in dependence of the neighbourhood angle (abscissa: 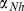) and communication status (*status I*: C,D; *status II*: E, *status III*: F). Closed circles, arithmetic means; vertical bars, SEMs (n = 7 data sets of 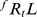) give the neighbours that were shimmering-active prior to (yellow: panels C_1_, E_1_) and after (red: D_1_, F_1_) the 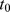 of the abdominal flipping of the *focus bees*. Fitting functions (blue: *PEAK* distribution pattern; green: *SINK* pattern) give the occurrence of shimmering-active neighbour bees (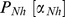 as predicted by the *directed-trigger* hypothesis (see Equations 4–6). Upward red and yellow arrows point to the 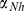 values at which the maximal numbers of active neighbours are predicted. (C_2_–F_2_) 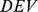 values (see Equations 8a,b) giving the deviation of the empirical 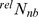 values from the expected probability 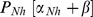 (Equation 7) with 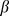 as the deviation angle of the fitting function; white curves, means; vertical bars, SEMs. Downward arrows point to the 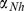 values at which the empirical distribution (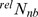) conforms to the *directed-trigger* hypothesis.

Theoretically, the simplest way of wave propagation is by spreading energy along a straight line by directed and non-stochastic processes. In shimmering, information is transferred by bridging information along chains of surface bees (in agreement with the *directed-trigger* hypothesis [7], [9]), where both, the *focus bees* and their *trigger neighbours*, are aligned perpendicular to the extension of the wave front. Although directedness in propagation has adaptive importance for shimmering [6], [9], [11]–[13], only less than 5% of agent bees (e.g., under 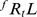) contribute to wave propagation in the main direction of a wave [9]. The present study confirms this apparent conundrum. An in-depth analysis was conducted (Fig. 10) with the data from one selected wave propagation direction (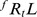) to test if the data confirms the *directed-trigger hypothesis* [7], [9].

#### PEAK and SINK concepts

The *directed-trigger hypothesis* [7], [9] predicts for every *focus bee* that their shimmering-active *neighbours* are distributed in angular sectors (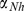) of the *near neighbourhood* according to Equation 4.
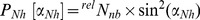
(4)where 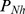 is the probability of the occurrence of shimmering-active neighbours (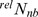) of a *focus bee* at the respective angle 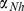 (for definition, see Fig. 10A).

The *directed-trigger hypothesis* postulates that in the *pre*-*stroke* phase of a *shimmering incident* the most shimmering-active neighbours (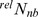) of *focus bees* are at the *ipsi-directional* side (where the wave is propagating from: see red arrows in Fig. 10A), and the least neighbours are present at the *contra-directional* side (where the wave is propagating to). For the *pre-stroke* phase, this hypothetical distribution of neighbours is described by Equations 5a,b and displayed in Fig. 10C_1_,E_1_, where blue curves show the maximal rates of shimmering neighbours 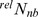 at the *ipsi-directional* side defined by 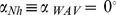 (under 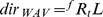). The distribution pattern shown in these curves is referred to as *PEAK* distribution pattern.
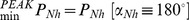
(5a)

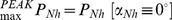
(5b)


In the *post-stroke* phase, the hypothetical distribution of shimmering-active neighbours conforms to the *SINK* distribution pattern, with a maximum occurrence of 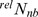 at the *contra-directional* side, and a minimum occurrence of 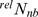 at the *ipsi-directional* side for 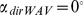 (see Equations 6a,b; green curves in Fig. 10 D_1_,F_1_).
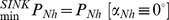
(6a)

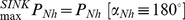
(6b)


### Matching the empirical data with the hypothetical PEAK and SINK patterns

The empirical 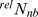 data were plotted against 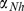 and the resulting distributions compared with the hypothetical *PEAK* and *SINK* distribution patterns. 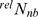 data of all three agent types (*bucket bridging*, *chain-tail* and *generator* agents) were tested for correspondence with the hypothetical *PEAK* and *SINK* distribution patterns by cross-correlation according to Equation 7.
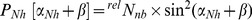
(7)Here, the neighbourhood angle 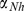 (for definition, see Figs. 1C_2_,10A) was altered by the deviation angle 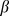 in steps of 45° (with −180°≤
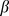
≤+180°) to identify the best match between the empirical data (displayed by full circles and vertical lines) and the *PEAK* and *SINK* curves. The hypothetical *PEAK* and *SINK* curves were normalized using the empirical differences between the 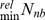 and 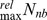 values for each agent type as reference (the normalized forms are indicated as {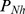} The sums of the square differences between the 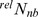 values with the {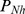} values were calculated for every angular step of neighbourhood [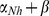] of the *focus bees*. The total deviation (*DEV*) over the total range of neighbourhood (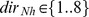) between the empirical values (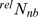) and the normalized *PEAK* and *SINK* values were determined by Equations 8a,b.
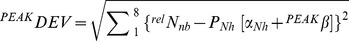
(8a)

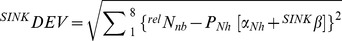
(8b)with 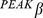 = 180° and 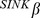 = 0°.

Their reciprocal values constitute the goodness of the fit 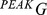 and 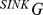 (Equations 9a,b) which can be tested using the χ^2^-test.
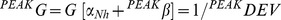
(9a)

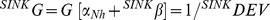
(9b)Furthermore, the probabilities 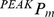 and 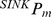 for the match between the empirical data and the hypothetical *PEAK* and *SINK* distribution patterns was calculated according to Equations 10a,b, explaining the coincidence of the agents' directional properties with the *directed-trigger hypothesis*.
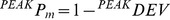
(10a)

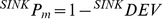
(10b)


The angular positions (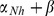), at which the *DEV* values exhibit a minimum (Fig. 10C_2_–F_2_), represent the maximal goodness (*max G*) of the fit (Fig. 10C_1_–F_1_). The resulting angular mismatch (MM) between the empirical data and the hypothetical *PEAK* and *SINK* distribution patterns was estimated by Equation 11, defined as the absolute deviation between the angle 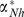 (the angle with the minimum value of *PEAK* or *SINK* patterns, see Fig. 10 C_1_–F_1_), and 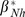 (the angle with the maximum goodness of the fit, see Fig. 10 C_2_–F_2_).
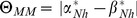
(11)Lastly, the level 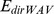 by which the different agent types contributed to the propagation of the main wave direction was estimated according to Equation 12.
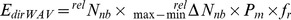
(12)where 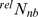 is the occurrence of shimmering-active neighbours of a *focus bee* in her *near neighbourhood*; 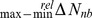 is the percentage of active neighbours in defined 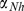 angles between maximal and minimal occurrences of 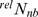; 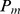 is the probability for the match between the empirical data and the hypothetical *PEAK* or *SINK* distribution patterns (Equations 10a,b); 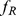 is the reinforcement factor with 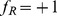 when the main wave direction is reinforced and 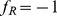 when the main wave direction is restrained.

#### Matching bucket-bridging agents

**Figure 11:**
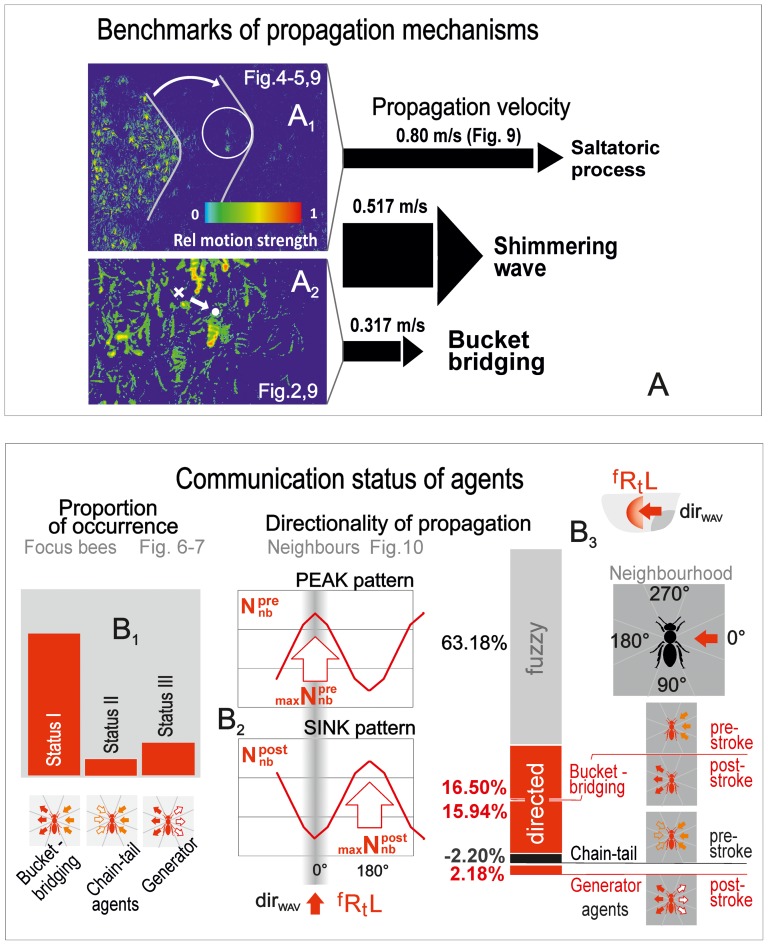
Summarization of results. (A) Assessment of benchmark values for both mechanisms of shimmering, the *saltatoric* wave propagation (panel A_1_, cf. Figs. 4–5,9) and *bucket bridging* (panel A_2_; Figs. 2,9). The lengths of the black arrows on the right side symbolize the propagation velocities of the partial processes (*saltatoric* processes and *bucket bridging*) and of *shimmering* as the mixed form of propagation. (B) Communication statuses of *focus bees* categorized in the *pre-stroke* and *post-* 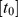-*stroke* phase by the recruitment level of their neighbours (nb), exemplified for the main wave direction 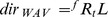; B_1_, the occurrence of the three types of agents (cf. Figs. 6–7); B_2_, schematics of the angular 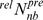 and 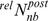 distributions of shimmering-active neighbours (*PEAK* and *SINK* distribution pattern, cf. Fig. 10) addressing the match with the *directed-trigger* hypothesis; B_3_, the impact of the three communication statuses on the control of directionality of the shimmering wave (cf. Fig. 10).

The *bucket-bridging* agents conformed to the *directed-trigger hypothesis* under *pre*-*stroke* and *post*-*stroke* conditions (Fig. 10 C_1_–D_1_) in different ways: the shimmering-active neighbours in the *pre-stroke* phase matched the *PEAK* distribution pattern four times more likely (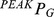 = 0.0061, χ^2^-test) than the *SINK* distribution pattern (Fig. 10 C_1–2_; Table S2: 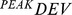 = 5.39%; 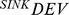 = 19.28%; 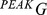 = 18.54; 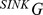 = 5.19; 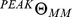 = 0°) and the factors calculated from equation 8 were 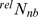 = 0.4716; 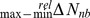 = 0.3697; 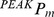 = 0.9461; 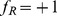). Conversely, in the *post-stroke* phase the shimmering-active neighbours matched the *SINK* distribution pattern rather than the *PEAK* distribution pattern (Fig. 10 D_1–2_, Table S2: 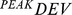 = 19.20%; 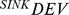 = 4.57%; 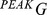 = 5.21; 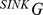 = 21.87, 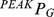 = 0.0014, χ^2^-test; 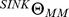 = 0°). The factors calculated from equation 8 were 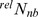 = 0.4101; 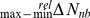 = 0.4073; 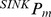 = 0.9543; 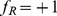. The total estimate of the influence of *bucket-bridging* agents to contribute to the wave propagation in the main shimmering direction was 32.44%, by adding up the 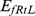 value in the *pre*-*stroke* phase (16.50%) and that in the *post*-*stroke* phase (15.94%; Fig. 11B, Table S2).

#### Matching chain-tail agents

The angular distribution of shimmering-active neighbours of the *chain-tail* agents agreed stronger with the *PEAK* than the *SINK* distribution pattern (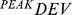 = 9.23%; 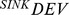 = 20.04%; 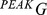 = 10.83; 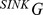 = 4.99; 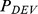 = 0.0459, 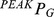 = 0.1421, χ^2^-test; Fig. 10 E_1–2_, Table S3), showing a small mismatch with the *PEAK*-pattern (Fig. 10E: 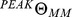 = 45° (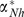 = 180°, 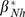 = 135°). This result shows that *chain-tail* agents can be identified by their match with the *PEAK* distribution pattern in the *pre-stroke* phase, and as they are the last agents in a chain they are the components that terminate the chain. Their contribution to shimmering (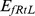 = −2.20%) can be considered as constraining the wave propagation, which is noted by the negative value of 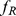 (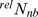 = 0.0556; 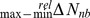 = 0.4781; 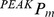 = 0.9077; 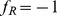; Fig. 11B, Table S3).

#### Matching generator agents

In their *post*-*stroke* phase, *generator* agents activate their neighbours. Their distribution pattern agreed twofold more with the *SINK* than the *PEAK* pattern (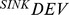 = 8.79%; 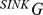 = 11.38; 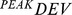 = 15.61%; 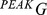 = 6.40; Table S4), although this difference was not significant (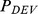 = 0.1669, 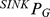 = 0.2380, χ^2^-test). There was a small mismatch with the *SINK* pattern (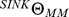 = 45° [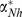 = 0°, 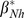 = 45°]; Fig. 10 F_1–2_) and the reinforcement of the main direction (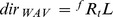) was 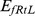 = 2.1793%; 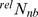 = 0.0675; 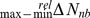 = 0.3538; 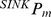 = 0.9121; 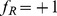; Fig. 11B, Table S4).

## Discussion

### Applying swarm models to giant honeybees

Shimmering in giant honeybees [1]–[9], [11]–[14] is an intricate communication behaviour based on *swarm intelligence* [18]–[20] with *emergent* [20]–[23] social waves. The term s*warm* [24]–[26] is used for the ability of aggregations of similar morphological units to self-organize [19]–[22], form patterns, store information, and reach collective decisions [26]–[28]. *Swarms* display fluidity and uniformity in response, which emerge in dynamic behavioural patterns, such as in fish schools [26]–[28], in the fast ephemeral rolling patterns of starlings flocks (in particular under predation by falcons or gulls [24], [29]), in the huddles of emperor penguins [30] or in groups of moving mammals [28], [31]. The simplest mathematical model of an animal *swarm* defines individual agents with a Lagrangian approach [24], [31]–[32], which have a gradient of repulsion and alignment around them following three rules: (a) *move in the same direction as your neighbours*, (b) *remain close to your neighbours*, and (c) *avoid collisions with your neighbours*. However, shimmering in giant honeybees does not match this *swarm* model: shimmering-active bees do not change their relative position in the bee curtain, and they move only parts of their body (Movies S1, S2, S3; [12]), although with the potential to generate directed moving patterns.

*Stigmergy* [34]–[35] (for summary and a broader definition see [36]) is another concept that may also help understanding the interaction between individual agents in shimmering. *Stigmergic* coordination stimulates behavioural responses through *traces* left in the environment. A shimmering wave affects all layers of the bee curtain mechanically [5], [7]–[8], [12] within tens of milliseconds (which can be considered as *traces*), thereby stimulating quiescent agent bees [7], [9] to join the wave.

For the analysis of shimmering, concentric zones of *neighbourhood* were implemented around each *focus bee*, similar to the Lagrangian swarm models [25], [32]–[33]. This concept allows the assessment of topological properties (cf. [37]) of the neighbours, such as the coordinates of the thoraces, and the strength and time of participation in the wave. Around 70% of shimmering-active bees (Fig. 7A_1–2_, *Status I*) act in a *bucket bridging*-like manner [9] while 88% of their immediate neighbours are also active (in the *pre-stroke* and *post-stroke* phases of *shimmering incidents*: Fig. 7A_3_, *Status I*). These agents contribute to the wave propagation in response to this mechanical commotion which mainly occurred in their *ipsi-directional* neighbourhood (where the wave came from). Their action also includes the release of Nasonov pheromone [38] that motivates defensive cohorts to stay rather than fly off and attack the enemy. They also stimulate their neighbours at their *contra-directional* side (towards which the wave is propagating) to join the abdominal flipping (Fig. 7C_1,2_, *Status I*).

On the other hand, a minority of about 15% (Fig. 7A_1_, *Status III*) of shimmering-active bees contribute to *saltatoric* wave propagation, and stimulate 6% (Fig. 7A_3_, *Status III*) of their neighbours to flip the abdomens. These *generator* agents start abdominal flipping before any *bucket-bridging* activity can be detected in their *near neighbourhood*, independent of the length of the discrimination window of <10 ff (Fig. 7B_3_). *Generator* agents are not prompted by their neighbours and therefore, have a lead role in decision-making by processing the visual cues of the threats, such as wasps in front of the nest, generating *daughter* waves. A *daughter* wave can merge with the steadily proceeding *parental* wave, forming a new wave front distally to the former *parental* wave front. Thus, the resulting *saltatoric* wave propagation is based on visual control which adds two new properties to the shimmering process [6]: it speeds up the wave front by a factor of up to three and allows the wave front to change direction rapidly with the potential that shimmering may even “follow” a threatening cue topologically on the nest (see Movies in [6], [39]).

### Goals of social waves

Social waves are hallmarks of animal aggregations. Besides giant honeybees, which display shimmering waves in response to wasps, birds and mammals [4]–[6], [12], social waves have also been described for avian flocks [24], [29], [37], [40]–[45], for the huddles of Emperor penguins [30], and for aggregations of humans in football stadiums [15]. A main question concerning social waves is whether they are advantageous for the aggregation, having received evolutionary function and conveying fitness benefits, or alternatively, whether they are simply a mere epiphenomenon of reactivity [43]. In the following, this question is discussed comparing the ephemeral rolling patterns in starlings and the shimmering waves in giant honeybees.

#### Waves in starling flocks

Starlings form flocks especially before they roost, to effectively maintain cohesion of the group, strongly supporting survival [37], [41]–[42]. Under predation they display spectacular ephemeral rolling patterns. Individual starlings may be kept informed in the flock about the progress and the state of such an ongoing “wave” by continuous inspection from the momentary vantage point. In reality, this information is restricted to a topological group of 6–7 neighbours [37]. Although an individual starling has some room to modify its decision to participate in the concerted flight manoeuvre, it cannot decline to conjointly move together with its neighbours. This constraint is less due to the potential danger of being eaten by the predator [29] but more due to the danger of collision [37], [41]–[42]. These rolling patterns display trains of pulses of optical density that propagate across the flock and are produced within the swarm body, mostly without affecting the swarm surface. The authors explain [29] that such pulses are formed in proximity to the bird of prey, mostly laterally, and are propagated typically away from it. These patterns may lead to *confusion* [6], [46] of the predator and encounter *dilution* [47] but the cause for their evolution is only poorly understood.

#### Shimmering waves

The goals of shimmering [5]–[13] in giant honeybees differ from the rolling waves of starling flocks [29], [37], [41]–[42] in the following ways: Shimmering waves are produced by stationary agents (Movies S1, S2, S3, S4, S5, S6). Most of the colony members, including those in subsurface layers or at the opposite comb side, continually receive information about the shimmering status mechanoceptically [7]–[8], [12]. Surface bees also release Nasonov pheromone [38], motivating others to participate in the wave. Shimmering giant honeybees may or may not join the wave, and if they join, they can determine when to start their contribution and at which strength [7], [9]. Both, starlings and giant honeybees have the potential to respond rapidly to changing visual cues, and in both cases directivity seems to be strongly controlled.

In giant honeybees, the directivity of *bucket bridging* was described previously [8]. In the present work, we show that the majority of shimmering-active bees (63.18%) do not contribute to the wave propagation in the main direction (Fig. 11B_3_). Of the other 36.82% of the shimmering-active bees which contribute to the directional control, 88.09% are *bucket-bridging* agents, 5.98% are *chain-tail* agents and 5.92% are *generator* agents (Table S2, S3, S4). *Generator* agents are less affected by the oncoming wave than the two other agent types (Fig. 7B_3_,10F), contributing less, and fuzzily, to wave direction control. This fuzziness of *generator* agents is important, because it enables rapid changes in wave direction in response to rapidly moving cues (cf. [39]).

### Why do giant honeybees speed up shimmering waves?

Taken together, the aspects discussed above appear to contribute to the *confusion* and *repellence* of predators [6], [12], [39], but they cannot explain why *saltatoric* propagation has evolved. Three possible explanations for this conundrum are discussed below: *saltatoric* propagation could reinforce (a) the recruitment of shimmering agents, (b) the repelling effect on predating wasps by directed visual patterns and (c) the *bottom-up* attention in vertebrate predators.

#### Saltatoric propagation reinforces recruitment

More agents are recruited when shimmering is accelerated above the “base” speed of *bucket bridging* (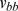 = 0.317 m/s) by *saltatoric* propagation (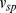 = 0.517 m/s) than during *bucket bridging* alone (Figs. 1–2,3–4,9). This increased recruitment is due to the generation of *daughter* waves, causing rapid, exponential growth of the visual pattern with a concomitantly greater repelling effect, which likely benefits the giant honeybee colony. This explanation is in agreement with previous findings [6] that more bees contribute to shimmering the faster and the nearer to the bee nest a preying wasp flies.

#### Saltatoric propagation increases the repelling effect on predating wasps by directed visual patterns

Giant honeybees have the capability to align the direction of their shimmering waves with the flight path of a preying wasp [6], [39]. In this prey-predator interaction both, the bees and the wasp emit signals and both respond to stimuli of their counterparts, whereby the honeybees can change the direction of their shimmering waves faster than the wasps can turn [39]. This asymmetry between bee and wasp is based upon the greater speed of wave propagation and the fuzziness in directional control by *saltatoric* propagation.

#### Saltatoric propagation may reinforce bottom-up attention in vertebrates

Giant honeybees display shimmering in response to wasps, but also to birds and mammals that approach the nest within distances of about 3 m (own observations). Shimmering produces repetitively moving circular areas, typically with a diameter of 20 cm or more. Mammals and birds may perceive these patterns as supernormal cues that represent moving, head-like structures [48], outwitting their perceptual systems. Such cues may release a startle response [48]–[49] in vertebrates based on *bottom up* (BU) attention [50]–[53]. *Bottom up* attention depends upon the properties of a sensory stimulus to capture full attention, such as a bright spot of colour, an area of sharp contrast, or a rapidly moving pattern, such as typically involved in shimmering [1]–[13].

In higher vertebrates, the retinal fovea is the main sensory interface for attention retrieval [50]–[51]. When a shimmering wave is imaged by the fovea under *covert* conditions (with fixated eyes), BU attention is likely stronger the greater the image of the shimmering wave is, until the image fully covers the fovea. The fovea receptors are most densely packed in the central 1–2°, and the maximal trace length of a moving object that crosses a central projection at the main acuity region of the fovea is less than 10° [50]–[51]. The trace length of a shimmering wave on a vertebrate fovea in the 200 ms of the *ascending* phase (Fig. 3B, [6]) will cover an angle of 10°, provided that circular patterns of 20 cm or more are produced and viewed from a distance of 1 m from the nest. In the *climax* phase of a shimmering wave (Fig. 3B), when the patterns move at a speed of 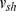 = 0.514 m/s (Fig. 9A) they will be viewed with a foveal path length of 8.154°, whereas mere *bucket-bridging* propagation (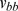 = 0.325 m/s) will affect only a path length of 5.400°.

## Summary and Conclusions

Characteristics and benchmark data of two propagation mechanisms (Fig. 11A) of shimmering, *bucket bridging* and the *saltatoric* processes were investigated. Combining both mechanisms speeds up the shimmering waves from 0.317 m/s (*bucket bridging* only) to 0.517 m/s, which shows that the *saltatoric* component, also associated with the generation of *daughter* waves, increases the overall propagation speed of shimmering by 40%. Three categories of shimmering-active surface bees were identified regarding their communication status: the *bucket-bridging*, *chain-tail* and *generator* agents. These agents comprise a characteristic proportion (Fig. 11B_1_) and a characteristic recruitment status with respect to their neighbours (Fig. 11B_2_), and they contribute differently to the directivity of the main shimmering wave (Fig. 11B_3_). Summarizing, the wave-like shimmering process in giant honeybees displays adaptive complexity, particularly regarding the generation and propagation of information, and is an impressive example of swarm intelligence [19]–[21]. Shimmering conforms to rules of *bucket-bridging* such as *linearity*, *continuity* and *graduality* [9] and involves an additional *saltatoric* strategy to speed up signal transmission, but also to provide a high level of fuzziness (Fig. 11B_3_) which may enable giant honeybee colonies to respond to rapidly changing threats [39].

## Supporting Information

Glossary S1 List of important terms, definitions and abbreviations used in this paper.
(DOCX)

Movie S1 In this episode two successive waves were generated by a dummy wasp that was moved from left to right (in the image). The episode started at f 1, but the playback session refers only to ff 550–650 (corresponding to 1,700 ms). The dummy wasp was moved 20 cm in front of the nest (shown at the left bottom corner of the image). The part of the nest displayed in the film corresponded to the area marked by the four yellow spots in Fig. 1A and comprised about 1,250 bees of the surface layer; the thorax of the *focus bee* selected for this film was marked in red. Left panel: black-and-white images, inverted to enhance the contrast of the abdomens; right panel: the same view but displayed as differential image with the motion strength in pseudo colours (with relative motion strength scaled from *blue* = 0.0 to *red* = 1.0; see rainbow scale on the right bottom side). Original recording speed: fps = 60 Hz; playback speed: fps = 60 Hz.
(AVI)

Movie S2 The same episode as in Movie S1, but zoomed for about 60 bees; original recording speed: fps = 60 Hz; playback speed: fps = 60 Hz.
(AVI)

Movie S3 The same episode as in Movie S1, playback speed: fps = 25 Hz (slow motion factor: 0.42 of original speed).
(AVI)

Movie S4 The same episode as in Movie S2, but with a playback speed of fps = 25 Hz as in Movie S3.
(AVI)

Movie S5 The same episode as in Movie S1, but with a playback speed of fps = 6 Hz (slow motion factor: 0.10 of original speed)
(AVI)

Movie S6 The same episode as in Movie S2, but with a playback speed of fps = 6 Hz as in Movie S5.
(AVI)

Table S1Accessory table to the Fig. 7 B–C, Fig. 8 A–C and Fig. 10 B with details of the regression functions regarding individual bees on the surface of the experimental giant honeybee nest B identified as *status I–III* agents (for definition, see text); tw, time window in [ff] at fps = 60 Hz; _all_dir, all four main directions of the spreading of the shimmering waves as selected in the paper: 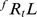, 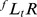, 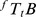, 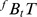; with *R,L,T,B* as *right, left, top, bottom*; abscissa and ordinate, the parameters used in the respective Figures/panels; coefficients of regressions (polynomials, exponential functions) are not detailed here; *number of cases* gives the number of *focus* bees or *neighbour* bees as evaluated in the data sets; *goodness of fit* (R^2^) regards the regression functions of mean values.(DOCX)

Table S2Survey of the data associated to Figs. 7,10 concerning agents of the *bucket-bridging* (*Status I*) type; experimental nest B (see Methods); ^a,b^, significant differences (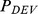
<0.01, χ^2^ test) within groups. The *hypothetical distributions* are normalized denotations of *PEAK* and *SINK* distribution patterns (see Results).(DOCX)

Table S3Survey of the data associated to Figs. 7,10 concerning agents of the *chain-tail* (*Status II*) type; experimental nest B (see Methods); ^a^ significant differences (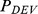 = 0.0459, χ^2^ test) within groups; na, not available data; the *hypothetical distributions* are normalized denotations of *PEAK* and *SINK* distribution patterns (see Results).(DOCX)

Table S4Survey of the data associated to Figs. 7,10, concerning agents of the *generator* (*Status III*) type; experimental nest B (see Methods); ^a^ non-significant differences (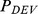 = 0.1669, χ^2^ test) within groups; na, not available data; the *hypothetical distributions* are normalized denotations of *PEAK* and *SINK* distribution patterns (see Results).(DOCX)
