# Supplementary material for: Speeding Up Social Waves. Propagation Mechanisms of Shimmering in Giant Honeybees
Source: PLoS One. 2014 Jan 27;9(1):e86315. doi: 10.1371/journal.pone.0086315 (PMC3903527; doi:10.1371/journal.pone.0086315)
Supplement: Glossary S1 — List of important terms, definitions and abbreviations used in this paper. (DOCX) [file pone.0086315.s001.docx]

### Glossary and Abbreviations

*Abdominal flipping, abdomen shaking:* The individual behaviour of giant honey bees when rising the abdomen and opening the gaps containing the Nasonov glands. Such traits are displayed under a series of behavioural contexts, primarily during *shimmering* and *flickering*.

*Agent bee*: Individual bees positioned at the surface of the nest which were identified by stereoscopic imaging, characterized by the moving of their thoraces. These movements were assessed as x- (horizontal directions), y- (vertical directions) and z- (directions towards and away from the comb) components at resolutions of fractions of a millimetre.

*Angle of neighbourhood:* The angular position of a *neighbour* bee relative to the position of the *focus* bee. The convention of this paper is to define the image-related angle *at the right side* of the *focus bee* as 0°, *at the bottom side* as 90°, *at the left side* as 180°, *at the top side* as 270°. (Fig. 1C_2_)

*Ascending phase*: The initial phase of a shimmering wave in which the number of synchronously shimmering-active surface bees and the number of *saltatoric* events increase. This phase lasts in the average 200-300 ms. (Fig. 3B)

$\beta$: This angle gives the deviation from the distribution predicted by the *directed-trigger* hypothesis and was altered in angular steps of 45° in the whole scope of application (‑180° ≤ $\beta$ ≤+180°) to calculate the match of the empirical data with the hypothetical *PEAK* or *SINK* distribution patterns. (Equations 7-9)

*Bee curtain*: The multiple-layered cover on both sides of the central comb of a giant honeybee nest with adult bees.

*Bottom-up (BU) attention*: This process is deployed very rapidly and depends only on the properties of a sensory stimulus which captures full attention, such as a bright spot of colour, an area of sharp contrast, the rapidness of a flash or the move of a pattern. The general concept of BU attention can be applied to vertebrates and expectedly also to insects.

*Bucket bridging* (in shimmering): The strategy of information transfer, by which agents (such as bees on the surface of the nest) are observed to contribute in the collective trait of shimmering by their abdominal flips along a linear chain of surface bees.

*Bucket bridging (≡ Status I) agents*: Agents who are driven by a number of shimmering-active neighbours in the *pre-stroke* phase and who pass on information to the neighbours at their *contra-directional* side in the *post-stroke* phase. This strategy of propagating information conforms to *bucket-bridging*. (Figs. 2,5)

*Cascading activity in shimmering:* Social waves such as shimmering are characterized by sequential activation of agents. In the *ascending* phase of a wave a single agent bee can activate more than one of her neighbours to do the same. This builds up a cascade with a gain of >1. Conversely, in the *descending* phase this gain factor is < 1 which means that an increasing cohort of shimmering-active agents fail to motivate others. (Figs. 4,5)

*Chain-tail agents*: Agent bees that participate in shimmering and are motivated by their nest mates in their *near neighbourhood* to do so. However, they are the last actively shimmering participant in the respective chain of surface bees. It seems as if the local wave process came about to stop after the abdomen flipping of these *chain-tail* agents, because for some time after their actions the *near neighbourhood* around them remained quiescent. (Fig. 5)

*Climax phase of a shimmering wave*: The phase of a shimmering wave in which the number of synchronously shimmering-active bees and the number of *saltatoric* events are maximized. This phase happens after the 200-300 ms of *ascending* phase before both wave parameters decrease again in the *descending* phase. (Fig. 3B)

*Communication status*: Every *focus bee* (which is, per definition, participates actively in shimmering by flipping the abdomen) can be characterized by one of three communication *statuses* (*I-III)* as defined by the time-space relations of her neighbours considering her *near neighbourhood* in the radius of < 40 mm in the *pre-stroke* and *post-stroke* intervals. (Fig. 5)

*Continuity in propagation*: Property of wave propagation if information is transferred over a chain of agents without interruption. In shimmering, the *bucket-bridging* process is *continuous*, but the *saltatoric* process is genuinely *discontinuous*. (Figs. 2,4)

*Daughter wave*: Specific cohorts of surface bees, the *generator* bees (≡ *Status III* agents), lift their abdomens in a solitary action, typically 30‑50 ms earlier than other participants, and more than 80 mm apart from the momentary position of the main front of a (*parental*) wave. Such agents may generate *daughter* waves which amalgamate with the proceeding *parental* wave. (Figs. 4,5)

*Descending phase*: The phase of a shimmering wave in which the number of synchronously shimmering-active surface bees is decreasing. It lasts 400-500 ms. (Fig. 3A)

${}_{\max-\min}^{rel}\Delta N_{nb}$ The percentage of active neighbours in defined $\alpha_{Nh}$ angles of *focus bees* between maxima and minima (${}_{\max-\min}^{rel}\Delta N_{nb} = {}_{\max}^{rel}N_{nb} - {}_{\min}^{rel}N_{nb}$ (Fig. 10)

*Direction of propagation of the wave* ($dir _{WAV}$, $\alpha_{WAV}$): Along with the angle of neighbourhood, the directions are defined respectively. We selected four discrete main directions for data evaluation. The direction f*rom Right to Left* (${}^{f}R_{t}L$) is defined as $dir _{WAV}$ = 0°, the direction f*rom Bottom to Top* (${}^{f}B_{t}T$) as $dir _{WAV}$ = 90° etc. (Fig. 1C_2_)

*DEV*: The deviation of the probability of the occurrence of shimmering-active neighbours (${}^{rel}N_{nb}$) of *focus* bees regarding the *pre*-stroke or *post*-stroke phase from the *PEAK* or *SINK* distribution patterns as predicted by the *directed-trigger* hypothesis. (Equations 8a,b; Fig. 10)

$d_{bb}$, $d_{E-R}$: Distance in mm between two adjacent shimmering-active surface bees which perform a singular step of information transfer. This happens in a chain of *bucket-bridging* (bb) cohorts between an emitter (E) agent bee who flips her abdomen upwards and a receiver (R) agent bee who senses this flip of her neighbour. (Figs. 2A,9D)

$E _{dir WAV}$: The reinforcement of the main direction in shimmering by the agents as estimated by the product of four recruitment factors. (Equation 12)

*f, ff*: Frames video recorded as a sequence of images at fps (frames per second) = 60 Hz.

*Focus bee (fb)*: An agent bee which has been selected (by manual inspection or by automated procedure) for analysis of the participation in shimmering. During a *shimmering incident* the time-positional parameters have been determined of herself and of her shimmering-active neighbours in two categories of *neighbourhood* (in the *near* and in the *far neighbourhood*). The time values of these neighbours were calibrated by the *focus-bee* specific starting time ($t_{0}$) of the abdomen flipping action. (Figs. 2-6).

*Frontline (of the shimmering wave)*: A shimmering process affects on its propagation path more and more *quiescent* agents. Therefore, the wave front is the transition zone between *quiescence* and motion. The mix of *synchronity*, and *cascading* / *saltatoric* activities of agents causes a specific width of this wave front on the surface of the nest. (Figs. 4-5)

$G [\alpha_{Nh}+\beta]$: Goodness of match of the empirical data with the hypothetical *SINK* and *PEAK* distribution patterns conforming to the *directed-trigger* hypothesis. (cf. Equations 8-9)

*Generator agents*: Agents who start a shimmering process prior to their neighbours. Thus, they are not triggered by their neighbours. These agents lack any shimmering-active neighbours in their *near neighbourhood* (< 40 mm) in the *pre-stroke* phase. (Figs. 4-6)

*Graduality in propagation in the shimmering process*: In shimmering, information transfer happens by the digital behaviour of flipping or non-flipping the abdomen. Additionally, the flipping action reveals also (analogue) information by its variable intensity. *Graduality* in spreading information in shimmering takes place if signal transmission is continued from one step to the next at the same energy level, if sequential recruitment of shimmering-active regards a similar number of agents or the similar flipping intensity per agent). (cf. Figs. 4,5)

$I_{dec}$: The time interval in ms within which an agent is allowed to *decide* whether or not to participate in the shimmering process. (Figs. 2,5,9C)

$k_{bb}$, $k_{sp}$: Weighting factors which quantify the impact of *bucket bridging* (bb) and *saltatoric* process (sp) in a shimmering wave. (Equations 3a,b; Fig. 9A)

*Linearity in propagation*: In shimmering, information transfer is associated with the propagation of the wave front and may happen in any direction over the surface of giant honeybee nests. This information transfer may take place over a chain of agents, by *bucket bridging* or by a *saltatoric process*, in a *linear* path. However, a *linear* propagation mode may be, not necessarily, *continuous* and *gradual*. (Figs. 2,4-5)

*Luminance (*$lum$*)*: The pixel-related values of brightness regarding the RGB channels range from $lum$ = 0 to $lum$ = 255.

*Mismatch angle* ($\Theta_{MM}$): The deviation between $\beta^{*}$ and $\alpha^{*}$: with $\beta^{*}$ as the angle of the maximum value of the goodness of the fit ($\beta^{*}$), between the empirical data (${}^{rel}N_{nb}$) with the hypothetical *PEAK* or *SINK* distribution patterns; $\alpha^{*}$ as the angle of the minimum values of the distribution patterns. (cf. Equations 8,11; Figs. 10 C-F)

*Mouth zone*: A giant honeybee nest constitutes of functional regions. Most time of the day the *bee curtain* displays a region, which represents an interface zone between outside and inside a nest, where the bees show locomotor behaviour, representing arriving and departing foragers, dancers, food-receivers and guard bees. (cf. Fig. 1A)

*Neighbourhood:* For every *focus bee*, two circular ranges of *neighbourhood* were defined: the *near neighbourhood* (< 40 mm) and the *far neighbourhood* (>40 mm,<100 mm). (Figs. 1C_2_,5,10)

*Neighbour bees (nb)*: For every *focus bee*, agents were identified as neighbours in the surface layer of the nest in two ranges of *neighbourhood*, provided these *neighbour* bees had actively participated in the wave. (Figs. 2,4-5)

$P_{Nh} [\alpha_{Nh}]$: The probability of occurrence of shimmering-active *neighbours* (${}^{rel}N_{nb}$) of a *focus bee* at the respective *angle of neighbourhood* $\alpha_{Nh}$ as predicted by the *directed-trigger* hypothesis. (Equation 4)

*Parental wave*: A shimmering episode at giant honeybee nests is built up by a wave-like process which can be described, at least if observed with naked eyes, as a seemingly *continuous* and uniform process. It exhibits a wave front which moves over the nest surface at a certain speed. Inspected in detail, this shimmering activity is composed of components which can be categorized in hierarchical way by *parental* and subordinate *daughter* processes. (Figs. 4-5)

*Participation in the wave:* Surface bees can be affected by a shimmering wave, but they may or may not participate in the ongoing process. The participation in a wave involves individual decisions. The agent bee decides whether she actively raises her abdomen, or remain in the passive status, by which she is moved by the oncoming wave. (cf. Fig. 2)

*PEAK pattern*: The probability of shimmering-active neighbours of *focus bees* is predicted by the *directed-trigger* hypothesis. It postulates that in the *pre-stroke* phase a *focus bee* the maximum number of shimmering-active neighbours (${}_{\max}^{PEAK}P_{Nh}$) is in the direction where the wave is propagating from and their minimum number (${}_{\min}^{PEAK}P_{Nh}$) is where the wave is propagating to. (Equations 5a,b; Figs. 10 C,E)

*Positional (x,y,z) parameters*: Every shimmering-active agent bee is identified by her thoracic positions (*x*,*y*,*z*) at the nest surface and by the ${}^{rel}M_{xy}$ values throughout the *shimmering incident*.

*Pre-stroke phase / (post-) stroke phase*: Every *focus bee* is defined by the starting time ($t_{0}^{fb}$) of her abdomen flipping action. The *pre-stroke phase* is defined as $t^{pre}$ < $t_{0}^{fb}$, and the (*post‑) stroke phase* as $t^{post}$ > $t_{0}^{fb}+\Delta t (1ff)$ (fps = 60 Hz). (Fig. 6).

${}^{rel}M_{xy}$: Parameter in image analysis quantifying the motion of an individual *agent bee* throughout the *shimmering incident*. It refers to the luminance changes in subsequent frames ($\Delta lum [f_{i-1},f_{i}]$) in a 60 x 60 px zone around the thorax of the selected agent bee. (Fig. 3A)

$r_{Nh}$: Radius of the *neighbourhood* of *focus bees*. (cf. Figs 1C_2_,5,10A)

${}^{rel}N_{nb}$: Relative number of *neighbour bees* (nb) around a *focus bee*. The reference (${}^{rel}N_{nb}$ = 1.00) was the total number of agents identified as shimmering-active *neighbour bees* in all shimmering waves evaluated (cf. Fig. 6)

*Saltatoric process:* Strategy of wave propagation set up by *generator* agents (Fig. 5 C_3_,D_3_) as a specific cohort of surface bees starting *daughter* waves in solitary actions. When a *daughter* wave amalgamates with a proceeding *parental* process the front line of the compound wave virtually *jumps* from the former status of the *parental* wave to the outer border of the *daughter* wave. Thus, the propagation of the front line of the compound *parental* wave is locally speeded up by a factor of two to five. (Figs. 3-4)

*Shimmering activity: Shimmering* behaviour is a main facet of defence behaviour in giant honeybees (*Apis dorsata*), whereby Mexican wave-like patterns are produced predominantly by bees at the nest surface, with the capability to repel predators from the bee nest. *Shimmering* bees flip their abdomen upwards in synchronized and cascading way (Movies 1-6).

*Shimmering incident*: The collective *shimmering* behaviour is built up by individual actions of surface bees which may participate in the wave actively or passively. An active contribution starts with a rapid upraise of the abdomen and ends after the individual resumes *quiescent* posture. Prior to the abdomen shaking phase, the agent is passively affected by the approaching wave front. The total session in which *passive* and *active* motion can be detected at an individual surface bee is termed as *shimmering incident*. (Fig. 3A)

*Shimmering flash*: For an external addressee a *shimmering* wave produces the impression of a visual pattern which is moving very fast and in a flash-like way. In detail, a *shimmering* wave develops in its *ascending* phase, within 200-300 ms, a head-like moving pattern which has the potential to induce *bottom-up attention* and a startle response in external observers.

*SINK pattern*: The probability of shimmering-active neighbours of *focus bees* is predicted by the *directed-trigger* hypothesis. It postulates that in the *post-stroke* phase a *focus bee* the minimum number of shimmering-active neighbours (${}_{\min}^{SINK}P_{Nh}$) occurs in the direction where the wave is propagating from and their maximum numbers (${}_{\max}^{SINK}P_{Nh}$) where the wave is propagating to. (Equations 6a,b, Fig. 10 D,F)

*Status I-III*: Communication statuses of a *focus* bee; they consider, as reference region, the *near neighbourhood* (< 40mm), and as reference time window, the *pre-stroke* and *post-stroke* intervals of 166.7 ms (corresponding to ±10 ff; fps = 60 Hz), before and after the onset of the abdomen flip which commences at the time $t_{0}$. A *focus* bee may be specified by one of three statuses and termed as *bucket-bridging*, *chain-tail* or *generator* agent. (Fig. 6)

*Surface layer*: The nest of giant honeybees constitutes of a central comb which is covered by a multi-layered curtain of honeybees. The nest mates at the roof (surface) layer of the nest have specific tasks for the entire collective of a honeybee colony. They protect the nest against mechanical perturbance such as caused by rain or wind, keep up homoeothermic conditions in the nest and control colony defence.

*Synchronity in shimmering activity:* In shimmering, cohorts of abdomen-flipping surface bees produce a Mexican-wavelike process causing for external addressees a visual pattern which moves across the nest surface. This *shimmering* pattern includes *cascading* principles, which control the propagation speed of a wave-like process, but comprises also synchronized recruitment to control the momentary intensity of the moving pattern. (Figs. 2,4-5; movies 1-6)

$t_{0}$: This time point defines time zero of a *shimmering incident*, the active participation of a surface bee in a shimmering wave. A *shimmering incident* starts one frame before motion is detected at an agent bee after a state of *quiescence*, followed by the abdomen lift. (Fig. 3A)

*Top-down (TD) attention*: This process filters sensory information in context of most behaviorally relevant events, and influences the perceptional process by *volitionarily* shifting attention towards the target. TD attention requires more effort to engage and is slower than the BU process.

*Trigger direction, trigger neighbour:* Every *focus* bee receives mechanical information from the oncoming wave by her adjacent *shimmering*-active neighbours. This neighbour who is nearest, and who flipped her abdomen at the shortest time interval prior to the abdomen flipping of the *focus bee*, was selected as *trigger neighbour*. The angular position of this neighbour relative to the position of the *focus bee* gives the *trigger angle* ($\alpha_{TRIG}$) or *trigger direction* ($dir _{TRIG}$). (For definition of angles, see Figs. 1,4,10)

$v_{bb}$, $v_{sp}$, $v_{sh}$, $v_{pw}$: Propagation velocities in *shimmering*, of *bucket bridging* (bb), of *saltatoric* propagation (sp), of the overall *shimmering wave* (sh) and of the *parental* wave (pw). (Figs. 2,3-5)
