# Supplementary material for: Speeding Up Social Waves. Propagation Mechanisms of Shimmering in Giant Honeybees
Source: PLoS One. 2014 Jan 27;9(1):e86315. doi: 10.1371/journal.pone.0086315 (PMC3903527; doi:10.1371/journal.pone.0086315)
Supplement: Table S1 — Accessory table to the Fig. 7 B–C, Fig. 8 A–C and Fig. 10 B with details of the regression functions regarding individual bees on the surface of the experimental giant honeybee nest B identified as status I–III agents (for definition, see text); tw, time window in [ff] at fps = 60 Hz; alldir, all four main directions of the spreading of the shimmering waves as selected in the paper: , , , ; with R,L,T,B as right, left, top, bottom; abscissa and ordinate, the parameters used in the respective Figures/panels; coefficients of regressions (polynomials, exponential functions) are not detailed here; number of cases gives the number of focus bees or neighbour bees as evaluated in the data sets; goodness of fit (R2) regards the regression functions of mean values. (DOCX) [file pone.0086315.s008.docx]

Table S1.

| ***Line*** | ***Figure Panel***  ***Symbol*** | ***Agent***  ***type /*** *Status* | ***Wave dir*** | ***Abscissa*** | ***Ordinate*** | ***Number of cases*** | ***Goodness***  ***R^2^*** |
| --- | --- | --- | --- | --- | --- | --- | --- |
|  | Fig 7 | ***Agents*** |  |  |  |  |  |
| 1 | A_1_ | *Focus bees* | $dir_{all}$ |  | ${}^{rel}N_{fb}$ | 13 678 |  |
| 2 | A_2_ | *Focus bees* | ${}^{f}R_{t}L$ |  | ${}^{rel}N_{fb}$ | 4 025 |  |
| 3 | A_3_ | *Neighbour bees* | ${}^{f}R_{t}L$ |  | ${}^{rel}N_{nb}$ | 29 248 |  |
|  | Fig 7 | ***Focus bees*** |  |  |  |  |  |
| 4 | B_1_ | *bucket bridging* | ${}^{f}R_{t}L$ | tw [ff] | ${}^{rel}N_{fb}$ | 2 759 (ff=10) | 0.9717 |
| 5 | B_2_ | *chain-tail* | ${}^{f}R_{t}L$ | tw [ff] | ${}^{rel}N_{fb}$ | 353 (ff=10) | 0.9884 |
| 6 | B_3_ | *generator* | ${}^{f}R_{t}L$ | tw [ff] | ${}^{rel}N_{fb}$ | 538 (ff=10) | 0.9878 |
|  | Fig 7 | ***Neighbour bees*** |  |  |  |  |  |
| 7 | C_1_ | *bucket bridging* | ${}^{f}R_{t}L$ | tw [-ff] | $N_{nb}^{pre}$ | 16 809 (ff=-10) | 0.9913 |
| 8 | C_2_ | *bucket bridging* | ${}^{f}R_{t}L$ | tw [+ff] | $N_{nb}^{post}$ | 19 333(ff=+10) | 0.9914 |
| 9 | C_3_ | *chain-tail* | ${}^{f}R_{t}L$ | tw [-ff] | $N_{nb}^{pre}$ | 2 056 (ff=-10) | 0.9995 |
| 10 | C_4_ | *generator* | ${}^{f}R_{t}L$ | tw [+ff] | $N_{nb}^{post}$ | 2 679 (ff=+10) | 0.9983 |
|  | Fig. 8 A | ***Neighbour bees*** |  |  |  |  |  |
| 11 | black | *bucket bridging* | $dir_{all}$ | $N_{nb}^{pre}/N_{nb}^{post}$ | ${}^{rel}N_{fb}$ | 3 472 | 0.9893 |
| 12 | red | *bucket bridging* | ${}^{f}R_{t}L$ | $N_{nb}^{pre}/N_{nb}^{post}$ | ${}^{rel}N_{fb}$ | 1 035 | 0.9933 |
|  | Fig. 8 B | ***Neighbour bees*** |  |  |  |  |  |
| 13 | black | *chain-tail* | $dir_{all}$ | $N_{nb}^{pre}$< 6 | ${}^{rel}N_{fb}$ |  | 0.9529 |
| 14 | black | *chain_tail* | $dir_{all}$ | $N_{nb}^{pre}$> 5 | ${}^{rel}N_{fb}$ |  | 0.9902 |
| 15 | red | *chain_tail* | ${}^{f}R_{t}L$ | $N_{nb}^{pre}$< 6 | ${}^{rel}N_{fb}$ |  | 0.6647 |
| 16 | red | *chain_tail* | ${}^{f}R_{t}L$ | $N_{nb}^{pre}$> 5 | ${}^{rel}N_{fb}$ |  | 0.9312 |
|  | Fig. 8 C | ***Neighbour bees*** |  |  |  |  |  |
| 17 | black | *generator* | $dir_{all}$ | $N_{nb}^{post}$< 5 | ${}^{rel}N_{fb}$ |  | 0.9984 |
| 18 | black | *generator* | $dir_{all}$ | $N_{nb}^{post}$> 4 | ${}^{rel}N_{fb}$ |  | 0.9952 |
| 19 | red | *generator* | ${}^{f}R_{t}L$ | $N_{nb}^{post}$< 5 | ${}^{rel}N_{fb}$ |  | 0.9999 |
| 20 | red | *generator* | ${}^{f}R_{t}L$ | $N_{nb}^{post}$> 4 | ${}^{rel}N_{fb}$ |  | 0,9586 |
|  | Fig. 10 | ***Focus bees*** |  |  |  |  |  |
| 21 | B |  | $dir_{all}$ | $c_{Mxy}$ | ${}^{rel}N_{fb}$ | 13 678 | 0.9991 |

Accessory table to the Fig.7 B-C, Fig.8 A-C and Fig.10 B with details of the regression functions regarding individual bees on the surface of the experimental giant honeybee nest B identified as *status I-III* agents (for definition, see text); tw, time window in [ff] at fps = 60 Hz; $dir _{all}$, all four main directions of the spreading of the shimmering waves as selected in the paper: ${}^{f}R_{t}L$, ${}^{f}L_{t}R$, ${}^{f}T_{t}B$, ${}^{f}B_{t}T$; with *R*, *L*, *T*, *B* as *right, left, top, bottom*; abscissa and ordinate, the parameters used in the respective Figures/panels; coefficients of regressions (polynomials, exponential functions) are not detailed here; *number of cases* gives the number of *focus* bees or *neighbour* bees as evaluated in the data sets; *goodness of fit* (R^2^) regards the regression functions of mean values.
