# Supplementary material for: Speeding Up Social Waves. Propagation Mechanisms of Shimmering in Giant Honeybees
Source: PLoS One. 2014 Jan 27;9(1):e86315. doi: 10.1371/journal.pone.0086315 (PMC3903527; doi:10.1371/journal.pone.0086315)
Supplement: Table S4 — Survey of the data associated to Figs. 7,10, concerning agents of the generator (Status III) type; experimental nest B (see Methods); a non-significant differences ( = 0.1669, χ2 test) within groups; na, not available data; the hypothetical distributions are normalized denotations of PEAK and SINK distribution patterns (see Results). (DOCX) [file pone.0086315.s011.docx]

Table S4.

|  |  | | **Status III / Generator / agents** | | | | | | | | | | | |  |  |  |  |  |  |  |  |  |  |  |  |  |  |  |
| --- | --- | --- | --- | --- | --- | --- | --- | --- | --- | --- | --- | --- | --- | --- | --- | --- | --- | --- | --- | --- | --- | --- | --- | --- | --- | --- | --- | --- | --- |
| **Line** | **Figure**  **Panel** | **Reference** | | **Equation** | **Template** | | | **Phase** | | | | | | **Impact [%]** |  |  |  |  |  |  |  |  |  |  |  |  |  |  |  |
|  |  | ***Shimmering-active neighbours*** ${}^{rel}N_{nb}$ ***[%]*** | | | |  | | |  | | |  | | | |  | | | | |  |  |  |  |  |  |  |  |  |
| 1 | na |  | |  |  | | | *pre-stroke* | | | | | | na |  |  |  |  |  |  |  |  |  |  |  |  |  |  |  |
| 2 | 7 A_3_ |  | |  |  | | | *post-stroke* | | | | | | 6.75 |  |  |  |  |  |  |  |  |  |  |  |  |  |  |  |
|  |  | ***Deviation of*** ${}^{rel}N_{nb}$ ***from hypothetical distributions [%]:*** ${}^{PEAK}DEV$***_,_*** ${}^{SINK}DEV$ | | | | | | | | |  | | | | |  | | | |  | | | | |  | | |  |  |
| 3 | na |  | | 3a | *PEAK* | | | *pre-stroke* | | | | | | na |  |  |  |  |  |  |  |  |  |  |  |  |  |  |  |
| 4 | na |  | | 3b | *SINK* | | | *pre-stroke* | | | | | | na |  |  |  |  |  |  |  |  |  |  |  |  |  |  |  |
| 5 | 10 F_1-2_ |  | | 3a | *PEAK* | | | *post-stroke* | | | | | | 15.61^a^ |  |  |  |  |  |  |  |  |  |  |  |  |  |  |  |
| 6 | 10 F_1-2_ |  | | 3b | *SINK* | | | *post-stroke* | | | | | | 8.79^a^ |  |  |  |  |  |  |  |  |  |  |  |  |  |  |  |
|  |  | ${}_{\max-\min}^{rel}\Delta N_{nb}$ **/ *Angular variance of*** ${}^{rel}N_{nb}$ ***[%]*** | | | | |  | | |  | | |  | | | |  | | | | |  |  |  |  |  |  |  |  |
| 7 | na | = ${}_{\max}^{rel}N_{nb}$ [0°] - ${}_{\min}^{rel}N_{nb}$ [180°] | |  | *PEAK* | | | *post-stroke* | | | | | | na |  |  |  |  |  |  |  |  |  |  |  |  |  |  |  |
| 8 | 10 F_1-2_ | = ${}_{\max}^{rel}N_{nb}$ [180°] - ${}_{\min}^{rel}N_{nb}$ [0°] | |  | *SINK* | | | *post-stroke* | | | | | | 35.38 |  |  |  |  |  |  |  |  |  |  |  |  |  |  |  |
|  |  | ***Probability by which*** ${}^{rel}N_{nb}$ ***matched with the { templates } [%]:*** $P_{m}=1-DEV$ | | | | | | | | | | | | | |  | | |  | | | | |  | | |  | | |
| 9 | na | ${}^{PEAK}P_{m}=1 - {}^{PEAK}DEV$ | |  | *PEAK* | | | *post-stroke* | | | | | | na |  |  |  |  |  |  |  |  |  |  |  |  |  |  |  |
| 10 | 10 F_1-2_ | ${}^{SINK}P_{m}=1 - {}^{SINK}DEV$ | |  | *SINK* | | | *post-stroke* | | | | | | 91.21 |  |  |  |  |  |  |  |  |  |  |  |  |  |  |  |
|  |  | ***Contribution in wave direction control [%]:*** $E_{fRtL}={}^{rel}N_{nb} \times{}_{\max-\min}^{rel}\Delta N_{nb}\times P_{m}\times f_{R}$ | | | | | | | | | | | | | |  | |  | | | | |  | | |  | | |  |
| 11 | na |  | |  | na | | | *pre-stroke* | | | | | | na |  |  |  |  |  |  |  |  |  |  |  |  |  |  |  |
| 12 | 10 F_1-2_ |  | |  | *SINK* | | | *post-stroke* | | | | | | 2.18 |  |  |  |  |  |  |  |  |  |  |  |  |  |  |  |
|  |  |  | |  |  | | |  | | | | | |  |  |  |  |  |  |  |  |  |  |  |  |  |  |  |  |

Survey of the data associated to Figs.7,10, concerning agents of the *generator* (*Status III*) type; experimental nest B (see Methods); ^a^ non-significant differences (P**_DEV_** = 0.1669, χ^2^ test) within groups; na, not available data; the *hypothetical distributions* are normalized denotations of *PEAK* and *SINK* distribution patterns (see Results).
